# Contrasting latitudinal patterns in phylogenetic diversity between woody and herbaceous communities

**DOI:** 10.1038/s41598-019-42827-1

**Published:** 2019-04-23

**Authors:** Jhonny C. Massante, Lars Götzenberger, Krista Takkis, Tiit Hallikma, Ants Kaasik, Lauri Laanisto, Michael J. Hutchings, Pille Gerhold

**Affiliations:** 1 Institute of Ecology and Earth Sciences, University of Tartu, Tartu, 51014 Estonia; 2 Institute of Botany, Academy of Sciences of the Czech Republic, CZ-37982 Trebon, Czech Republic; 3 Institute of Agricultural and Environmental Sciences, Estonian University of Life Sciences, Tartu, 51006 Estonia; 4 School of Life Sciences, University of Sussex, Falmer, Brighton, Sussex, BN1 9QG UK

**Keywords:** Community ecology, Evolutionary ecology, Macroecology, Phylogenetics, Speciation

## Abstract

Although many studies have shown that species richness decreases from low to high latitudes (the Latitudinal Diversity Gradient), little is known about the relationship between latitude and phylogenetic diversity. Here we examine global latitudinal patterns of phylogenetic diversity using a dataset of 459 woody and 589 herbaceous plant communities. We analysed the relationships between community phylogenetic diversity, latitude, biogeographic realm and vegetation type. Using the most recent global megaphylogeny for seed plants and the standardised effect sizes of the phylogenetic diversity metrics ‘mean pairwise distance’ (SES_mpd_) and ‘mean nearest taxon distance’ (SES_mntd_), we found that species were more closely-related at low latitudes in woody communities. In herbaceous communities, species were more closely-related at high latitudes than at intermediate latitudes, and the strength of this effect depended on biogeographic realm and vegetation type. Possible causes of this difference are contrasting patterns of speciation and dispersal. Most woody lineages evolved in the tropics, with many gymnosperms but few angiosperms adapting to high latitudes. In contrast, the recent evolution of herbaceous lineages such as grasses in young habitat types may drive coexistence of closely-related species at high latitudes. Our results show that high species richness commonly observed at low latitudes is not associated with high phylogenetic diversity.

## Introduction

The latitudinal diversity gradient (LDG), which shows a pattern of decreasing species richness from the equator towards the poles^1,2^ is one of the earliest recorded and most striking global biogeographical patterns. Many hypotheses have been proposed to explain the LDG^3–6^. At the core of most of these hypotheses is the issue of the way in which evolution has shaped this pattern. For example, the time-integrated species-area hypothesis^7^ and the tropical conservatism hypothesis^8^ suggest that species diversity decreases with latitude because tropical biomes are larger and older than temperate biomes. Moreover, many currently extant clades originated in the tropics^9,10^, and therefore species in these clades have had more time, and a larger area in which to diversify, than would have been the case if they had originated at higher latitudes. It is widely believed that, following their evolution under tropical conditions, few of the species in these clades, or their descendants, subsequently migrated far from their region of origin^8^.

Using information about currently co-existing species to study the way in which evolution might have shaped the LDG makes the assumption that the species from which ecological communities are composed themselves provide valuable evolutionary information^11^. In particular, in addition to differing in species composition and richness, communities also display considerable differences in the evolutionary origin - i.e. the phylogenetic relatedness - of their constituent species. This aspect of biodiversity is reflected in community phylogenetic diversity. Two communities may have similar species diversity, but differ considerably in phylogenetic diversity because of differences in the evolutionary history of their constituent species. A community consisting of closely-related species has a low phylogenetic diversity compared to a community consisting of the same number of distantly-related species. Measuring and comparing phylogenetic diversity in different plant communities therefore offers the possibility of interpreting latitudinal diversity gradients not only on the basis of present-day ecological processes, but also by considering the role of evolutionary drivers including speciation and extinction.

Localized speciation causing low phylogenetic diversity has been observed repeatedly in a variety of plant communities. For example, neo-endemism, i.e. recent localized evolutionary radiation, is responsible for low phylogenetic diversity in Amazonian white-sand forests^12^. Similarly, localized radiations have resulted in low phylogenetic diversity in communities of the Qinghai-Tibetan Plateau, Central Asia^13^. The recently radiated lineages Restionaceae and Ericaceae have produced communities with low phylogenetic diversity in South African semi-arid shrublands^14^. Recent and rapid radiations in the genus *Eucalyptus*^15^ have resulted in low phylogenetic diversity in some Australian subtropical rainforest communities^16^. In contrast, low extinction rates, which result in long-term accumulation of distantly-related lineages, can result in communities with high phylogenetic diversity^17,18^. For example, coexistence of distantly-related plant lineages with resprouting ability in response to fire has promoted high phylogenetic diversity in low latitude Brazilian savannah communities throughout the last 8 million years^19^, and geographic isolation coupled with trait conservatism in distantly-related lineages has resulted in high phylogenetic diversity in the mountain forests of Malesia^20^.

Apart from speciation and extinction events, macroecological factors such as climate and regional stability can influence community phylogenetic diversity. Climate is a strong driver of phylogenetic diversity of angiosperm tree assemblages at regional scales^21,22^ and across the globe^23,24^ because of its tendency to select closely related species. Phylogenetically diverse tree assemblages are associated with long-term climatic stability^25^ and environmental heterogeneity^26^. Although various evolutionary and macroecological factors are major drivers of species diversity, little attention has been given to the way in which these factors combine to influence the LDG. To our knowledge, no comparative studies have been conducted on plant communities, and although studies have been carried out on specific taxonomic groups, including woody angiosperms^24^, amphibians^27^ and mammals^28^, no global-scale study has been undertaken to examine variation in phylogenetic diversity across a wide taxonomic range of plant communities.

**Figure 1:**
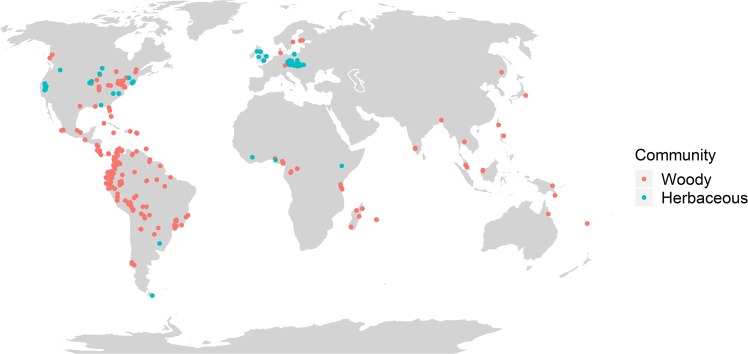
Locations of plant communities included in this study. Red circles = woody communities (N = 459); blue circles = herbaceous communities (N = 589). Multiple data points may be superimposed. The global map was plotted using the Robinson projection.

In this study, we conduct a global meta-study to investigate patterns of phylogenetic diversity in contemporary plant communities along the latitudinal gradient from the tropics to polar regions (Fig. 1), in the context of evolutionary and macroecological processes. Specifically, we examine the relationships between the phylogenetic diversity of woody and herbaceous plant communities and latitude, biogeographic realms^29^, and vegetation type. We analysed woody and herbaceous communities independently because the presence of distinct growth forms (i.e. woody and herbaceous species) throughout the latitudinal gradient depends on the responses of many species in these categories to climatic variables that are correlated with latitude^30^. We calculated the standardised effect size of the mean pairwise distance (SES_mpd_), and standardised effect size of the mean nearest taxon distance (SES_mntd_) across the species recorded in each community. The first index measures phylogenetic distances across the whole phylogenetic tree by averaging all species pairwise distances, whereas the latter measures the phylogenetic distance at a shallower level, namely between closest relatives. Both indices have been shown to be independent of species richness when the number of species is held constant during randomizations^31^. Although these indices reflect “community phylogenetic structure” or “phylogenetic relatedness” of coexisting species, we use the term “phylogenetic diversity” of communities hereafter for simplicity.

## Results

Results of all tests are listed in Tables 1 and 2. Parameter estimates of the top-ranked linear mixed effects models are also shown in the Supplementary Information (Supplementary Tables S1, S2). In response to the wide range of values in latitude, there was considerable variation in phylogenetic diversity across the communities in our dataset, with SES_mpd_ ranging from −10.50 to 9.54, and SES_mntd_ ranging from −7.82 to 3.56. Model selection indicated the best models for each measure of phylogenetic diversity in each of the two types (i.e. woody, herbaceous) of communities (Table 1). The contribution of fixed effect variables (i.e. marginal R^2^) to the variability in phylogenetic diversity in the top-ranked models generally amounted to approximately half of the variability explained by both fixed and random effects variables for SES_mpd_ in woody communities and SES_mntd_ in herbaceous communities. It corresponds to 25% and 28% of the total variance explained by the models respectively. The fixed effect variables in the top-ranked models of SES_mpd_ in herbaceous communities and SES_mntd_ in woody communities explained 13% and 11% of the total variance respectively (Table 1). The inclusion of sampling unit size in the models did not affect the relationships in any community type (Table 2, Supplementary Tables S1, S2).

**Table 1:** Ranking of the linear mixed effects models for linear and quadratic (latitude^2^) effects of latitude and other studied variables on community phylogenetic diversity (standardised effect size of the mean pairwise distance – SES_mpd_, and standardised effect size of the mean nearest taxon distance – SES_mntd_) in woody and herbaceous communities.

Community type	Phylogenetic diversity	Model	logLik	AICc	∆AIC_c_	AIC_w_	R^2^_m_	R^2^_c_
Woody	SES_mpd_	**latitude** + **latitude**^**2**^ + **size** + **vegetation**	**−947.968**	**1916.4**	**0.00**	**0.400**	**0.25**	**0.46**
		latitude + latitude^2^ + size + realm	**−**945.322	1917.5	1.03	0.238	0.28	0.47
		latitude + latitude^2^ + size	**−**950.605	1917.5	1.10	0.230	0.23	0.45
		latitude + latitude^2^ + size + realm + vegetation	**−**943.783	1918.6	2.22	0.132	0.29	0.48
	SES_mntd_	**latitude** + **latitude**^**2**^ + **size**	**−843.544**	**1703.4**	**0.00**	**0.850**	**0.11**	**0.45**
		latitude + latitude^2^ + size + vegetation	**−**843.302	1707.1	3.69	0.135	0.11	0.45
		latitude + latitude^2^ + size + realm	**−**842.417	1711.7	8.24	0.014	0.12	0.44
		latitude + latitude^2^ + size + realm + vegetation	**−**842.286	1715.7	12.25	0.002	0.12	0.44
Herbaceous	SES_mpd_	**latitude** + **latitude**^**2**^ + **size** + **realm** + **vegetation**	**−997.599**	**2019.7**	**0.00**	**0.805**	**0.13**	**0.48**
		latitude + latitude^2^ + size + vegetation	**−**1002.130	2022.6	2.83	0.195	0.13	0.51
		latitude + latitude^2^ + size + realm	**−**1008.466	2039.4	19.65	0.000	0.08	0.48
		latitude + latitude^2^ + size	**−**1012.197	2040.6	20.90	0.000	0.06	0.49
	SES_mntd_	**latitude** + **latitude**^**2**^ + **size** + **realm** + **vegetation**	**−861.604**	**1747.7**	**0.00**	**0.762**	**0.28**	**0.38**
		latitude + latitude^2^ + size + vegetation	**−**865.881	1750.1	2.32	0.238	0.15	0.40
		latitude + latitude^2^ + size + realm	**−**882.025	1786.5	38.76	0.000	0.11	0.36
		latitude + latitude^2^ + size	**−**886.475	1789.2	41.45	0.000	0.01	0.41

The models are sorted by corrected Akaike information criterion value (AIC_c_), with log likelihood (logLik), difference in AIC_c_ from the top-ranked model (∆AIC_c_), model weight (AIC_w_), marginal R^2^ (R^2^_m_), and conditional R^2^ (R^2^_c_). Size = sampling unit size; vegetation = vegetation type (closed, open, semi-open); realm = biogeographic realm (Afrotropical, Australasian, Indo-Malayan, Nearctic, Neotropical, Palearctic)^29^. Study identification was included as a random effect variable in all models.

**Table 2:** Estimated effects of the variables in the top-ranked mixed effects models on community phylogenetic diversity (standardised effect size of the mean pairwise distance – SES_mpd_, and standardised effect size of the mean nearest taxon distance – SES_mntd_) in woody and herbaceous communities.

Community type	Phylogenetic diversity	Top-ranked model	Variables	Coefficient	F-value	p-value
Woody	SES_mpd_	latitude + latitude^2^ + size + vegetation	Intercept	0.2437	2.9568	0.0868
			Latitude	—	37.6293	<**0.0001**
			Linear term	1.0241***	—	—
			Quadratic term	0.5636***	—	—
			Size	0.2065	3.1324	0.0781
			Vegetation	—	2.6106	0.0758
			Open	−3.3202*	—	—
			Semi-open	–0.3985	—	—
	SES_mntd_	latitude + latitude^2^ + size	Intercept	−2.7331	538.3517	<0.0001
			Latitude	—	13.9199	<**0.0001**
			Linear term	0.5869***	—	—
			Quadratic term	0.0522	—	—
			Size	0.1664	3.2111	0.0745
Herbaceous	SES_mpd_	latitude + latitude^2^ + size + realm + vegetation	Intercept	13.1456	4.3958	0.0365
			Latitude	—	5.2857	**0.0053**
			Linear term	0.5858	—	—
			Quadratic term	−1.4841**	—	—
			Size	−0.0714	0.9973	0.3184
			Realm	—	2.8835	0.0541
			Nearctic	−14.3492*	—	—
			Neotropical	−12.4259*	—	—
			Paleactic	−13.1274	—	—
			Vegetation	—	21.4457	<**0.0001**
			Open	−1.3713***	—	—
	SES_mntd_	latitude + latitude^2^ + size + realm + vegetation	Intercept	−1.3252	0.1169	0.7325
			Latitude	—	3.4684	**0.0318**
			Linear term	5.9833	—	—
			Quadratic term	11.0210	—	—
			Size	−0.0380	0.4748	0.4910
			Realm	—	2.8352	0.0569
			Nearctic	1.9078	—	—
			Neotropical	0.8520	—	—
			Paleactic	0.5757	—	—
			Vegetation	—	41.9209	<**0.0001**
			Open	−1.3868***	—	—

Variables were tested with ‘anova.lme’ function from nlme package^72^, using the marginal significance of each fixed effect variable coefficient when all other fixed effects variables are present in the model. Size = sampling unit size; vegetation = vegetation type (closed, open, semi-open); realm = biogeographic realm (Afrotropical, Australasian, Indo-Malayan, Nearctic, Neotropical, Palearctic)^29^. Significance of individual coefficients are flagged with stars: *p < 0.05, **p < 0.01, ***p < 0.001. Estimates for biogeographic realms refer to the deviation from Afrotropical, and for vegetation type to the deviation from closed. All parameter estimates of the top-ranked models are shown in Supplementary Tables S1 and S2. Study identification was included as a random effect variable in all models.

**Figure 2:**
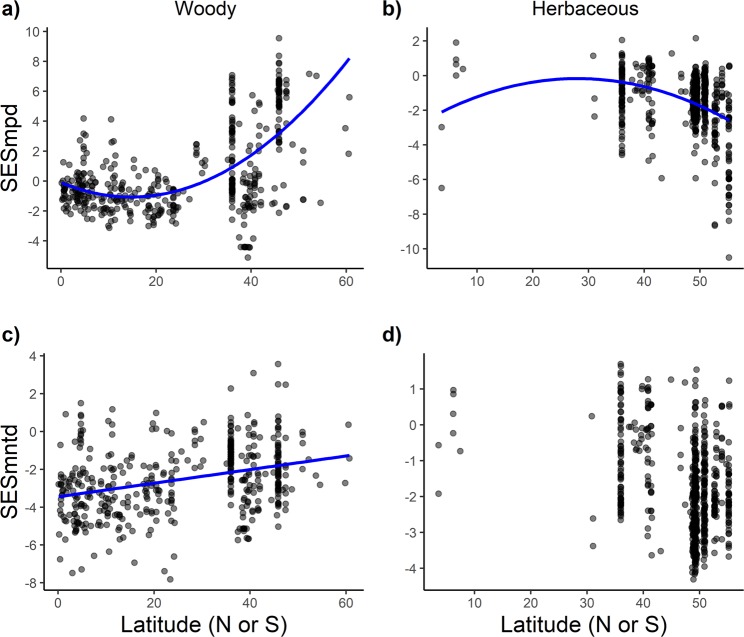
Effect of latitude on phylogenetic diversity of plant communities. Standardised effect size of mean pairwise distance (SES_mpd_) in woody (**a)** and herbaceous (**b**) communities. Standardised effect size of mean nearest taxon distance (SES_mntd_) in woody (**c**) and herbaceous (**d**) communities. Multiple data points may be superimposed. Lines are based on the predicted values from the top-ranked models (see Table 2).

There were contrasting patterns of phylogenetic diversity in woody and herbaceous communities along the latitudinal gradient. In woody communities there were positive relationships between both measures of phylogenetic diversity and latitude (Fig. 2a,c). SES_mpd_ showed a quadratic relationship, whereas SES_mntd_ showed a positive linear relationship with latitude, with species being more closely-related at low latitudes in both cases. Vegetation type affected the relationship between SES_mpd_ and latitude in woody communities (Supplementary Table S1 and Supplementary Fig. S1).

In contrast, the coefficient of the quadratic term for latitude in the top-ranked model of SES_mpd_ had a negative value (Supplementary Table S2) which means that phylogenetic diversity was lower at high latitudes than at intermediate latitudes (Fig. 2b). SES_mntd_ did not show a significant relationship with latitude (Fig. 2d, Supplementary Table S2). Vegetation type was the most important determining variable in the models for both measures of phylogenetic diversity: phylogenetic diversity was lower in open vegetation types than in closed vegetation types (Table 2, Supplementary Fig. S2b,d). Biogeographic realm was a significant determinant of SES_mpd_: phylogenetic diversity was higher in communities from the Afrotropical realm than in those from the Nearctic and Neotropical (Table 2, Supplementary Table S2, Supplementary Fig. S2a).

Excluding gymnosperms from woody communities resulted in a negative linear relationship between SES_mpd_ and latitude (Supplementary Results, Supplementary Tables S3, S4, Supplementary Fig. S3a), and a positive linear relationship between SES_mntd_ and latitude (Supplementary Results, Supplementary Tables S3, S4, Supplementary Fig. S3b).

## Discussion

In accordance with several recent studies^22,24,26–28,32–35^, our results demonstrate that evolutionary and macroecological mechanisms contribute to explaining differences in the phylogenetic diversity of contemporary plant communities along the latitudinal gradient. In this study, data from a wide range of plant communities, distributed from the tropics to extreme polar regions, displayed significant effects of latitude on phylogenetic diversity. While local assembly processes acting at small scales may also influence community phylogenetic structure^36–39^, the inclusion of sampling unit size as a covariate in the models did not affect the latitudinal trends in phylogenetic diversity that we report (Table 2, Supplementary Tables S1, S2). This suggests strongly that the trends we report are unlikely to be altered by processes acting at very small scales.

Woody species were more closely-related in communities located at lower latitudes. The variance explained by fixed effect variables was 25% and 11% for SES_mpd_ and SES_mntd_ respectively. The higher level of explanation in the case of SES_mpd_ is probably due to latitude being a major factor, coupled with vegetation type along the latitudinal gradient. Low phylogenetic diversity in communities at low latitudes probably arises because most woody lineages originated in warm climates such as those found in humid tropical regions^10,40–42^ in the Early Cretaceous (*c*. 146–100 myr^43,44^). In contrast to the results reported here, previous studies have demonstrated a pattern of lower phylogenetic diversity in woody communities located in colder and/or dryer regions^21–24^. This difference probably arises because the species lists used in our study involved complete lists of all woody species, including gymnosperms (110 gymnosperm species were included in the dataset in total). In contrast, earlier studies^21–24^ omitted gymnosperms because records of these species were rare in the data and consequently generated exceptionally long evolutionary branches in the phylogeny. We included gymnosperms because at the global scale they dominate many communities and occupy large areas of many regions around the globe (e.g. the Podocarpaceae and Araucariaceae in the Southern Hemisphere, and the Pinaceae in the Northern Hemisphere).

Excluding gymnosperms from woody communities revealed a negative relationship between SES_mpd_ and latitude, which is in accordance with results from previous studies, and a positive relationship between SES_mntd_ and latitude. A probable explanation for the negative relationship when gymnosperms are excluded, is that harsh conditions at high latitudes exclude angiosperm taxa that have evolved in warmer climates and favour taxa adapted to colder temperatures. The positive relationship between SES_mntd_ and latitude probably arises from the fact that the division between angiosperms and gymnosperms deep in the phylogeny does not affect the relationships at the tips of the phylogeny. Gymnosperms were the most abundant plant species until the emergence of the angiosperms in the Late Cretaceous (*c*. 94–66 myr^45^), and global cooling at the end of the Eocene would have led to polewards expansion of temperate forests^46^. This might have created new regeneration niches for gymnosperms. Thus, the presence of phylogenetically distant gymnosperm species may have made a significant contribution to the high phylogenetic diversity we observed in woody communities located at high latitudes. Given that ecosystems at low latitudes are a major source of woody species, and that most gymnosperm species have physiological advantages over the angiosperms in cold, nutrient-poor environments^47,48^, the tendency for higher phylogenetic diversity at higher latitudes could result both from the combination of *in situ* speciation of woody angiosperms at low latitudes, and from niche conservatism of woody gymnosperms at high latitudes.

In contrast to the results for woody communities, we found that phylogenetic diversity in herbaceous communities decreased towards extreme latitudes, and that this effect was influenced mainly by vegetation type. Low phylogenetic diversity at high latitudes accords with the relatively recent origin of temperate and boreal climates and habitats^7^ compared to the tropics, and with the young age of some lineages present at high latitudes, such as the grasses. Herbaceous lineages may be able to cope with low availability of resources at high latitudes through niche evolution^49^. Additionally, most herbaceous species at high latitudes are well-adapted to freezing conditions because of their ability to produce and replace short-lived aboveground tissue at low cost^50^.

Our results indicate that phylogenetic diversity has a weaker relationship with latitude in herbaceous communities than in woody communities. The weaker relationships in herbaceous communities could result from the concentration of numerous studies exhibiting a high level of variability in phylogenetic diversity in Central Europe (Fig. 1), even though the spatial aggregation of those studies did not result in any significant spatial autocorrelation. Species tended to be more closely-related in herbaceous communities at high latitudes relative to moderate latitudes, but the latitudinal trend is more pronounced when calculated from the whole phylogenetic tree (SES_mpd_) than when calculated among closest relatives (SES_mntd_) (Fig. 2b, Supplementary Table S2). Low SES_mpd_ values at high latitudes are probably a result of recent rapid diversification in several herbaceous lineages following the rise of angiosperm-dominated herbaceous flora in open habitats^51^ during global cooling in the Late Cretaceous (*c*. 83–66 myr^52^) and Eocene-Oligocene (*c*. 51–33 myr^53^). For example, the Ranunculaceae, which originated as a forest clade, rapidly diversified *c*. 83 myr into 11 herbaceous lineages in mid- to high latitudes^51^. Likewise, the family Valerianaceae originated in the Himalayas of Asia in the early Cenozoic (66–40 myr^54,55^) and subsequently dispersed into temperate latitudes and higher elevation habitats, eventually reaching the Andes^55^, where strong diversification occurred^56^. Despite the large number of herbaceous communities analysed in our study, the results need to be interpreted with caution because of spatial limitation in the distribution of the original studies. Herbaceous communities were restricted both in latitude (98% were from latitudes above 30°N) and in the climate space occupied (mostly temperate climate, Supplementary Fig. S5). Our results also showed that vegetation type might be a major determinant of phylogenetic diversity in herbaceous communities, as shown in Table S2, and by the difference in the marginal R^2^ values between the top-ranked models for SES_mpd_ and SES_mntd_ (13% and 28% respectively). As in the case of SES_mntd_, latitude is not a significant predictor.

Our results show that open communities were generally less phylogenetically diverse than closed communities. This agrees with results of Lososova *et al*.^57^, which showed that the phylogenetic diversity of species pools in grasslands and other open habitats was lower than that in forests in Central Europe. Lososova *et al*.^57^ attributed this to the historical age of the vegetation types: grasslands were formed during the late Tertiary, with a limited number of lineages having enough time to adapt to the open conditions, whereas high phylogenetic diversity in forests results from continuous accumulation of lineages since the Mesozoic. Proches *et al*.^14^ also found lower phylogenetic diversity in vegetation types that have undergone recent radiation, such as grassland, fynbos and Nama-Karoo, compared to evolutionarily older subtropical scrub vegetation in South Africa.

We only found a significant effect of biogeographical realm on SES_mpd_ in herbaceous communities. This is surprising because different biogeographic realms are known to support the development of distinct patterns of biodiversity as a result of historical factors^58^. The failure to observe effects of biogeographic realm in several cases may have been caused by communities from some realms, such as the Afrotropics, being relatively poorly represented in our dataset. Alternatively, our results indicate that the latitudinal gradient in phylogenetic diversity may be a general macroecological trend across biogeographical realms.

Although most lineages exhibit higher species richness at lower latitudes^1,6^, our results demonstrate that if phylogenetic diversity is measured using indices that are independent of species richness, it cannot be uncritically assumed that the same pattern will be observed^28,59^. Instead, the results of this study suggest that the impacts of the mechanisms proposed to explain latitudinal diversity gradients may differ between woody and herbaceous growth forms. Several speciation and extinction events have affected lineages during evolutionary time, causing a variety of patterns of phylogenetic diversity to develop in plant communities, with the important consequence that lineages with different growth forms have responded idiosyncratically to changing environmental circumstances^60^.

This study adds further insight into the cradle/museum debate about the causes of regional diversity^18^, although the data do not allow us to test these hypotheses directly. Nevertheless, our results support the proposal that the latitudinal diversity gradient may arise from different regions acting as either cradles or museums for lineages, with communities containing closely-related species (i.e. with low phylogenetic diversity) arising in regions acting as evolutionary cradles, and communities consisting of more distantly-related species (i.e. with high phylogenetic diversity) being found in regions acting as museums, in which conditions promote the preservation of a high degree of evolutionary diversity. Testing these hypotheses would require data on speciation and extinction rates in lineages of both woody and herbaceous growth forms.

We have demonstrated that plant communities consisting of species that have arisen through different evolutionary lineages, and are dominated by species with different growth forms, display contrasting patterns of phylogenetic diversity across the latitudinal gradient. These findings reinforce the view that both evolutionary and ecological processes should be taken into consideration in future efforts to explain latitudinal diversity gradients.

## Methods

### Plant community data

We compiled a global data set using the libraries of ISI Web of Knowledge for papers published between 1945 and August 2016, and JSTOR for papers published between 1700 and 1945. We searched for simultaneous occurrence of the key words “community”, “vegetation”, “species list” and “plant”, to find papers with full species lists for plant communities (Supplementary Dataset S1). We considered all sampling methods used by the authors of the selected original studies. We use the term “community” to refer to locally coexisting species that were recorded in sampling units (e.g. plots, transects, regional surveys) ranging in size from <1 m^2^ in herbaceous communities, to >1 km^2^ in woody communities. Number of sampling units ranged from 1 to 367 in woody communities and from 1 to 160 in herbaceous communities. We separated the dataset into woody communities composed only of woody species (shrubs and trees), and herbaceous communities composed only of herbaceous species, based on the sampling information provided by the authors of the original studies. Communities composed of both woody and herbaceous species were excluded from the dataset. We also excluded studies with only partial species lists (e.g. lists only for dominant species, or only for some taxonomic groups). We excluded aquatic and urban communities, and communities with fewer than three species to avoid including anthropogenic communities, e.g. arable fields, plantations. The latitudinal range of the locations of the studied communities ran from 0° to a maximum of 60.67°N of the equator and a maximum of 54.79°S of the equator (Fig. 1).

We also included 223 woody communities from Alwyn Gentry’s dataset^61^, available from http://salvias.net/pages/database_info.php. In total, this resulted in 459 woody communities in which a total of 8,753 species were recorded, and 589 herbaceous communities in which a total of 1,847 species were recorded (Supplementary Dataset S1 and Supplementary References S1). Woody communities included forests, scrublands and savannas, whereas herbaceous communities included the herbaceous layer of forests, grasslands, meadows, salt marshes, outcrops and dunes (Supplementary Dataset S1).

We extracted data from the database WorldClim^62^ for mean annual temperature (hereafter temperature) and annual precipitation (hereafter precipitation) at the locations of every community. These data were extracted at a spatial resolution of 30 arc seconds.

### Phylogenetic data

A phylogeny of species was obtained based on the most up to date megaphylogeny for seed plants^63^, which comprises 79,881 taxa. We standardised the species names in our dataset according to The Plant List using the R package ‘Taxonstand’^64^. For those taxa identified to genus level or with misspelled names, we standardised their names manually according to The Plant List. We then used the R function S.PhyloMaker^65^ to link the species names in our dataset with those in the megaphylogeny, and the scenario 3 approach^65^ to add species to the phylogeny. We used this same approach for taxa identified to genus level. Scenario 3 adds missing taxa (e.g. genera or species) to the phylogeny within the taxa with known branch lengths, in a similar way to the approach implemented in Phylomatic and BLADJ^66^. We pruned our complete phylogeny to create two trees which included either (i) only those species recorded in the woody communities or (ii) only those species recorded in the herbaceous communities in our dataset. These two trees were then used as reference lists from which phylogenetic diversity could be calculated for every community in the dataset.

Phylogenetic diversity, which often depends on species richness, can be quantified in several ways^31^. We used the index ‘mpd’, which calculates phylogenetic distances across the whole phylogenetic tree by averaging all species pairwise distances, and the index ‘mntd’, which calculates phylogenetic distances at a shallower level, between the most closely-related pairs of species. These indices are opposite in sign to the net relatedness index (NRI) and nearest taxon index (NTI), respectively^36,66^, and they measure phylogenetic diversity between species at different depths in the phylogenetic tree. To produce indices of phylogenetic diversity that were independent of species richness, we calculated the standardised effect sizes of both indices (SES_mpd_ and SES_mntd_), by comparing the observed community phylogenetic diversity to the null distribution of randomly assembled communities with equal richness. Negative values of SES_mpd_ and SES_mntd_ indicate lower phylogenetic diversity than expected under the assumption of the null model, whereas values greater than zero indicate higher phylogenetic diversity than predicted by the null model. We calculated phylogenetic diversity using the R software program^67^ and the package ‘picante’^68^.

### Statistical analysis

Latitude is widely recognized as a surrogate for environmental variables including temperature^6^ and precipitation^69^. To determine whether to include both temperature^6^ and precipitation in further analyses, we first analysed the correlation between latitude and both mean annual temperature and annual precipitation, using Pearson’s product moment correlation. We found strong correlations in both cases (Supplementary Table S5), and therefore used only latitude in further analyses, in combination with other non-correlated variables (see below). The distribution of woody and herbaceous communities in the climate space is shown through a Whittaker biomes plot^70^, produced using the function ‘whittaker_base_plot’ in the package ‘plotbiomes’^71^ (Supplementary Fig. S5).

We analysed the patterns of community phylogenetic diversity (SES_mpd_ and SES_mntd_) along the latitudinal gradient in woody and herbaceous communities using linear mixed-effect models (function ‘lme’) in the ‘nlme’ package^72^. For each of the two categories of community (woody and herbaceous), we built alternative mixed-effect models with different combinations of the following fixed effects variables: latitude, biogeographical realm^29^ (Afrotropical, Australasian, Indo-Malayan, Nearctic, Neotropical, Palearctic) and vegetation type. As we expected latitude to have an effect on phylogenetic diversity, but were unable to predict whether the relationship would be linear, we included both linear and quadratic terms in the models. The effect of vegetation type on community phylogenetic structure was examined by extracting information about vegetation type from each of the published studies (e.g. forests, dunes, scrublands) and reclassifying them as either closed (forest communities), open (grassland, meadow, salt marsh, outcrop and dune communities), and semi-open (savanna and scrubland communities). To account for the influence of differences in sampling unit size, we included this as a covariate in the fixed effects variables of the models. All continuous fixed effects variables were centered and standardised to have zero mean and unit variance before parameter estimation in order to make them comparable within models. We fitted models with all combinations of fixed effects variables, excluding interactions between them using maximum likelihood (“ML”) to allow comparison between models with different predictors. We then ranked all models based on the Akaike information criterion corrected for small sample size (AICc). The most parsimonious models were refitted using restricted maximum likelihood (“REML”). The fixed effects variables in the most parsimonious models were then compared with the ‘anova.lme’ function in ‘nlme’^72^, using the marginal significance of each fixed effect variable when all other fixed effects variables were present in the model. Additionally, we used two types of R^2^ (marginal and conditional R^2^) proposed by Nakagawa & Schielzeth^73^ for mixed-effect models, using the ‘MuMIn’ package^74^. Marginal R^2^ represents the variance explained by the fixed effects variables, whereas the conditional R^2^ represents the variance explained by the whole model, including both fixed and random effects variables^73^.

Spatial autocorrelation is commonly found in ecological data observed across geographical space^75^. To account for heterogeneous and spatial aspects of the dataset, we used ‘study’ as a random factor in all models. In addition, as our dataset involved a clustered spatial arrangement of communities (in particular those in Central Europe and Northwestern South America), we fitted all models with an additional term describing the within-group correlation structure using the ‘corExp’ function in ‘nlme’ package^72^. We also plotted the normalised residuals of the top-ranked models on the global map (Supplementary Fig. S6).

In order to examine how the inclusion of gymnosperm species affected the pattern of phylogenetic diversity in woody communities along the latitudinal gradient, we ran mixed-effects models with gymnosperm species excluded (Supplementary results, Supplementary Tables S3, S4, Supplementary Figs S3, S4). All figures were produced in the package ggplot2^76^. Statistical analyses were performed in R software program version 3.4.3^67^.

## Supplementary information

Supplementary Information

Dataset 1

R Scripts

## Data Availability

All data analysed during this study are included in this article and in its Supplementary Dataset and Supplementary Information files. R scripts including species name standardisation, calculation of phylogenetic diversity indexes, and all statistical analyses are also available as Supplementary Information.
