# Publisher Correction: Contrasting latitudinal patterns in phylogenetic diversity between woody and herbaceous communities

**DOI:** 10.1038/s41598-019-44332-x

**Published:** 2019-05-22

**Authors:** Jhonny C. Massante, Lars Götzenberger, Krista Takkis, Tiit Hallikma, Ants Kaasik, Lauri Laanisto, Michael J. Hutchings, Pille Gerhold

**Affiliations:** 1 Institute of Ecology and Earth Sciences, University of Tartu, Tartu, 51014 Estonia; 2 Institute of Botany, Academy of Sciences of the Czech Republic, CZ-37982 Trebon, Czech Republic; 3 Institute of Agricultural and Environmental Sciences, Estonian University of Life Sciences, Tartu, 51006 Estonia; 4 School of Life Sciences, University of Sussex, Falmer, Brighton, Sussex BN1 9QG UK

Correction to: *Scientific Reports* 10.1038/s41598-019-42827-1, published online 23 April 2019

A supplementary file containing R scripts was omitted from the original version of this Article. This has been corrected in the HTML version of the Article; the PDF version was correct at time of publication.

