# Supplementary material for: Contrasting latitudinal patterns in phylogenetic diversity between woody and herbaceous communities
Source: Sci Rep. 2019 Apr 23;9:6443. doi: 10.1038/s41598-019-42827-1 (PMC6478853; doi:10.1038/s41598-019-42827-1)
Supplement: Supplementary file 1 — Supplementary Information [file 41598_2019_42827_MOESM1_ESM.pdf]

Supplementary Information

Contrasting latitudinal patterns in phylogenetic diversity between woody and herbaceous communities

Jhonny C Massante*[1], Lars Götzenberger[2], Krista Takkis[3], Tiit Hallikma[3], Ants Kaasik[1], Lauri Laanisto[3], Michael J Hutchings[4] and Pille Gerhold[1]

[1]Institute of Ecology and Earth Sciences, University of Tartu, Tartu 50410, Estonia

[2]Institute of Botany, Academy of Sciences of the Czech Republic, CZ-37982 Trebon, Czech Republic

[3]Institute of Agricultural and Environmental Sciences, Estonian University of Life Sciences, Tartu 51014, Estonia

[4]School of Life Sciences, University of Sussex, Falmer, Brighton, Sussex BN1 9QG, UK

*Corresponding author: Institute of Ecology and Earth Sciences, University of Tartu, Tartu 51014, Estonia. Phone: +372 737 5853; E-mail: jhonny.massante@ut.ee.

1. Supplementary tables, figures, and results

Table S1. Parameter estimates in the top-ranked linear mixed effects models on community phylogenetic diversity (standardised effect size of the mean pairwise distance – SES_{mpd}, and standardised effect size of the mean nearest taxon distance – SES_{mntd}) in woody communities. Size = sampling unit size; vegetation = vegetation type (closed, open, semi-open). Estimates for vegetation type refer to the deviation from closed. Study identification was included as a random effect variable in all models.

Phylogenetic diversity					
SES_{mpd}	Top-ranked model	Coefficient	Std.Error	t-value	p-value
	Intercept	0.2437	0.1417	1.7195	0.0868
	**Latitude**	1.0241	0.1341	7.6356	< 0.0001
	**Latitude^2**	0.5636	0.1161	4.8512	< 0.0001
	Size	0.2065	0.1167	1.7698	0.0781
	Vegetation	-	-	-	-
	**Open**	-3.3202	1.5189	-2.1858	0.0298
	Semi-open	-0.3985	0.6016	-0.6623	0.5084
	Spatial autocorrelation				
	Range	0.0000			
	Nugget	0.0000			
	Random effect (Standard Deviation)				
	Intercept	1.0818			
	Residual	1.7196			
SES_{mntd}	Intercept	-2.7331	0.1177	-23.2024	< 0.0001
	**Latitude**	0.5869	0.1112	5.2743	< 0.0001
	Latitude^2	0.0522	0.0947	0.5512	0.5820
	Size	0.1664	0.0929	1.7919	0.0745
	Spatial autocorrelation				
	Range	0.0001			
	Nugget	0.0000			
	Random effect (Standard Deviation)				
	Intercept	1.0408			
	Residual	1.3062			

Table S2. Parameter estimates in the top-ranked linear mixed effects models on community phylogenetic diversity (standardised effect size of the mean pairwise distance – SES_{mpd}, and standardised effect size of the mean nearest taxon distance – SES_{mntd}) in herbaceous communities. Size = sampling unit size; realm = biogeographic realm (Afrotropical, Nearctic, Neotropical, Palearctic); vegetation = vegetation type (closed, open). Estimates for biogeographic realms refer to the deviation from Afrotropical, and for vegetation type to the deviation from closed. Study identification was included as a random effect variable in all models.

Phylogenetic diversity	Top-ranked model	Coefficient	Std.Error	t-value	p-value
SES_{mpd}	Intercept	13.1456	6.2698	2.0966	0.0365
	Latitude	0.5858	0.5683	1.0309	0.3030
	**Latitude^2**	-1.4841	0.5083	-2.9195	0.0036
	Size	-0.0714	0.0715	-0.9986	0.3184
	Biogeographic realm	-	-	-	-
	**Nearctic**	-14.3492	6.2379	-2.3003	0.0294
	**Neotropical**	-12.4259	5.9130	-2.1014	0.0451
	Palearctic	-13.1274	6.4064	-2.0491	0.0503
	Vegetation	-	-	-	-
	**Open**	-1.3713	0.2961	-4.6309	< 0.0001
	Spatial autocorrelation				
	Range	0.0002			
	Nugget	0.0000			
	Random effect (Standard Deviation)				
	Intercept	1.2370			
	Residual	1.2570			
SES_{mntd}	Intercept	-1.3252	3.8753	-0.3419	0.7325
	Latitude	0.2467	0.3412	0.7230	0.4699
	Latitude^2	0.4544	0.3274	1.3881	0.1657
	Size	-0.0380	0.0551	-0.6891	0.4910
	Biogeographic realm	-	-	-	-
	Nearctic	1.9078	3.8741	0.4924	0.6264
	Neotropical	0.8520	3.6470	0.2336	0.8170
	Palearctic	0.5757	3.9437	0.1459	0.8850
	Vegetation	-	-	-	-
	**Open**	-1.3868	0.2141	-6.4746	< 0.0001

Spatial autocorrelation	
Range	0.0001
Nugget	0.0000
Random effect (Standard Deviation)	
(Intercept)	0.5435
Residual	1.0199

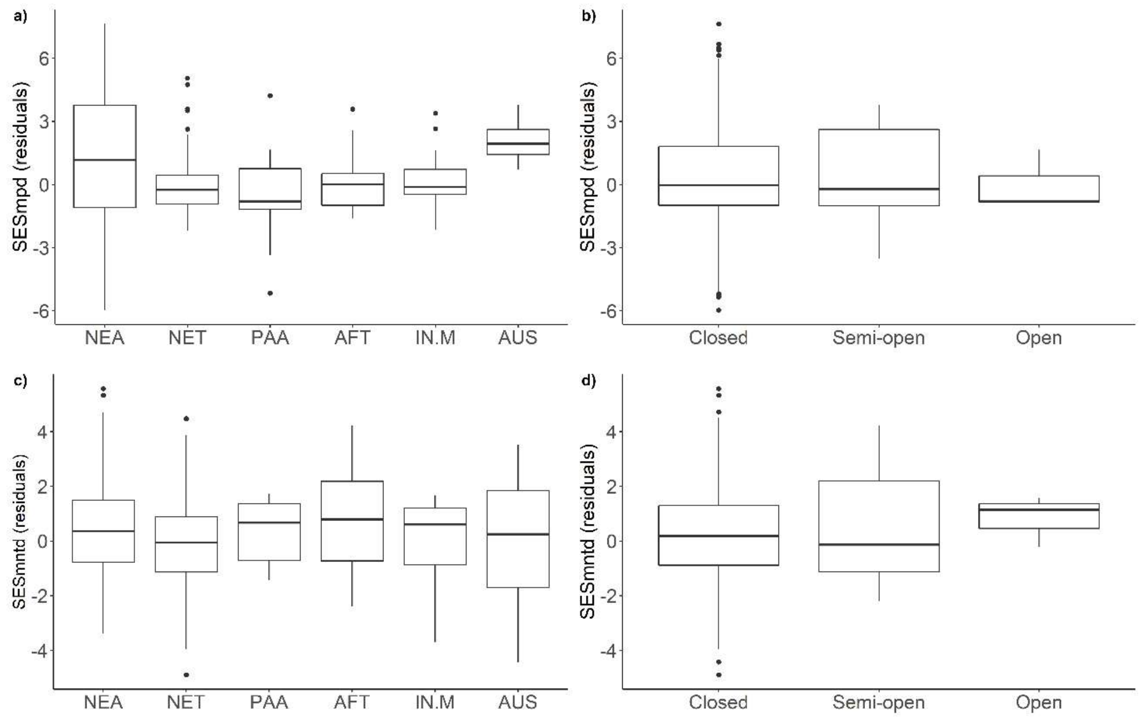

Figure S1. Partial residuals plots for **(a, c)** biogeographic realms and **(b, d)** vegetation types on phylogenetic diversity of woody communities in the top-ranked models (see Table S1 for parameter estimates) while holding all other fixed effects constant. SES_{mpd} = standardised effect size of the mean pairwise distance. SES_{mntd} = standardised effect size of the mean nearest taxon distance. NEA. = Nearctic (N = 221); NET = Neotropical (N = 175); PAA = Palearctic (N = 11); AFT = Afrotropical (N = 32); IN.M = Indo.Malayan (N = 16); AUS = Australasian (N = 4). Closed (N = 438); Semi-open (N = 18); Open (N = 3). Horizontal lines inside the boxes = median values, boxes = 25% and 75% quartiles, vertical lines = 10% and 90% percentiles, dots = outliers. Note that biogeographic realms are not included in both top-ranked models whereas vegetation types are included only in the top-ranked model with SES_{mpd} as response variable.

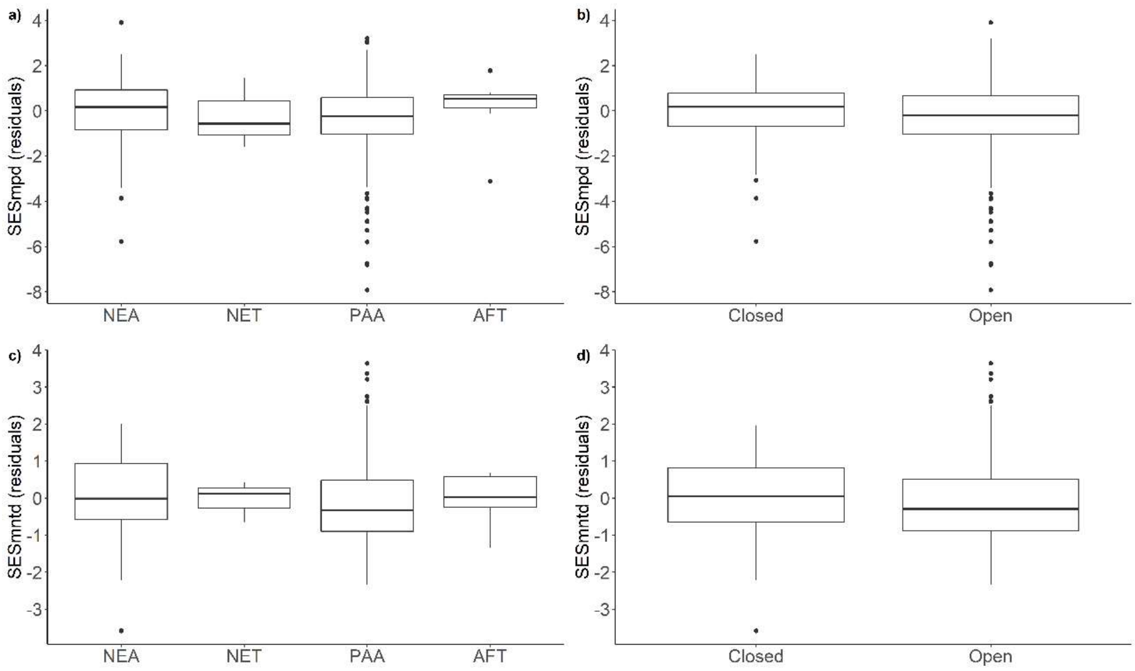

Figure S2. Partial residuals plots for **(a, c)** biogeographic realms and **(b, d)** vegetation types on phylogenetic diversity of herbaceous communities in the top-ranked models (see Table S2 for parameter estimates) while holding all other fixed effects constant. SES_{mpd} = standardised effect size of the mean pairwise distance. SES_{mntd} = standardised effect size of the mean nearest taxon distance. NEA. = Nearctic (N = 131); NET = Neotropical (N = 3); PAA = Palearctic (N = 448); AFT = Afrotropical (N = 7). Closed (N = 88); Open (N = 501). Horizontal lines inside the boxes = median values, boxes = 25% and 75% quartiles, vertical lines = 10% and 90% percentiles, dots = outliers.

Supplementary results

Phylogenetic diversity in woody communities excluding gymnosperm species

We excluded gymnosperm species from the phylogenetic tree using the function ‘drop.tip’ available in the ‘ape’ package[1]. We then ran the phylogenetic analysis described in the methods section (see main text) to produce the estimates of standardised effect size of phylogenetic diversity using the functions ‘ses.mpd’ and ‘ses.mntd’ available in the ‘picante’ package[2]. We included a spatial autocorrelation term in all models. We fitted models with all combinations of covariates with maximum likelihood, and then ranked the models based on the Akaike Information Criterion corrected for small sample size (AICc, Supplementary Table S3). We refitted the most parsimonious models using restricted maximum likelihood (“REML”) and calculated parameter estimates (Supplementary Table S4).

The results show that woody communities without gymnosperm species tend to be composed by closely-related angiosperm species towards the poles as showed by previous studies3–6 when the phylogenetic distance is calculated across the whole phylogenetic tree (SES_{mpd}, Supplementary Figure S3a). At shallower phylogenetic levels (SES_{mntd}), however, the communities with gymnosperm species excluded remain phylogenetically over-dispersed towards the poles (Supplementary Figure S3b), similar to the communities with gymnosperm species included (Supplementary Figure 2c). The low marginal R2 of SES_{mntd} indicates that a great deal of variation has not been explained either by the top-ranked model or by other models (Supplementary Table S3). We speculate that other variables that were not included in our study might also influence the latitudinal patterns of angiosperm plant communities. These might include local extinction of closely-related species and/or speciation events in distantly-related clades. Ecological factors such as

habitat heterogeneity and long dispersal may also play a role. There are also differences in SES_{mpd} between biogeographic realms, but not in SES_{mntd} (Supplementary Figure S4). A probable explanation for the different pattern along the latitudinal gradient and between biogeographical realms is that when gymnosperms are excluded, angiosperm species at high latitudes represent a group filtered out by harsh environments that exclude tropical taxa and favour taxa adapted to cold temperatures[7]. Supplementary figure S1 shows that the biogeographic realm Nearctic has higher SES_{mpd} values than other biogeographic realms. The Nearctic realm is located mostly at colder latitudes, where gymnosperms are favoured over angiosperms[8]. When data for gymnosperms are excluded from the analysis, this pattern changed. Instead, Nearctic realm had SES_{mpd} values lower than or not distinguishable from those of other realms (Supplementary Figure S4). The opposite latitudinal gradient in SES_{mntd}, i.e. at shallower phylogenetic level probably arises from the fact that the split between angiosperms and gymnosperms deep in the phylogeny does not affect the relationships at the tips of the phylogeny. These differences reinforce the novelty of our study which advocates the inclusion of a wide taxonomic range of plant communities in studies of phylogenetic diversity in the context of evolutionary and macroecological processes.

Table S3. Ranking of the linear mixed effects models for linear and quadratic (latitude^2) effects of latitude and other studied variables on community phylogenetic diversity (standardised effect size of the mean pairwise distance – SES_{mpd}, and standardised effect size of the mean nearest taxon distance – SES_{mntd}) in woody communities without gymnosperm species. The models are sorted by corrected Akaike information criterion value (AIC_c), with log likelihood (logLik), difference in AIC_c from the top-ranked model (ΔAIC_c), model weight (AIC_w), marginal R^2 (R^2_m), and conditional R^2 (R^2_c). Size = sampling unit size; vegetation = vegetation type (closed, open, semi-open); realm = biogeographic realm (Afrotropical, Australasian, Indo-Malayan, Nearctic, Neotropical, Palearctic). Study identification was included as a random effect in all models.

Phylogenetic diversity	Model	logLik	AICc	ΔAIC_c	AIC_w	R^2_m	R^2_c
SES_{mpd}	**latitude + latitude^2 + size + realm**	**-820.245**	**1667.3**	**0.00**	**0.676**	**0.26**	**0.70**
	latitude + latitude^2 + size + realm + vegetation	-819.558	1670.2	2.90	0.159	0.26	0.70
	latitude + latitude^2 + size	-827.163	1670.7	3.32	0.129	0.23	0.70
	latitude + latitude^2 + size + vegetation	-826.331	1673.2	5.83	0.037	0.23	0.70
SES_{mntd}	**latitude + latitude^2 + size**	**-806.313**	**1629.0**	**0.00**	**0.826**	**0.06**	**0.47**
	latitude + latitude^2 + size + vegetation	-805.828	1632.2	3.21	0.166	0.06	0.47
	latitude + latitude^2 + size + realm	-805.944	1638.7	9.78	0.006	0.06	0.46
	latitude + latitude^2 + size + realm + vegetation	-805.488	1642.1	13.14	0.001	0.06	0.47

Table S4. Parameter estimates in the top-ranked linear mixed effects models on community phylogenetic diversity (standardised effect size of the mean pairwise distance – SES_{mpd}, and standardised effect size of the mean nearest taxon distance – SES_{mntd}) in woody communities without gymnosperms. Size = sampling unit size; realm = biogeographic realm (Afrotropical, Nearctic, Neotropical, Palearctic. Estimates for biogeographic realms refer to the deviation from Afrotropical. Study identification was included as a random effect variable in all models.

Phylogenetic diversity	Top-ranked model	Coefficient	Std.error	t-value	p-value
SES_{mpd}	Intercept	-1.9004	0.5179	-3.6694	0.0003
	**Latitude**	-0.9296	0.3041	-3.0563	0.0025
	**Latitude^2**	0.3520	0.1430	2.4612	0.0147
	Size	-0.0925	0.0907	-1.0202	0.3088
	Biogeographic realm	-	-	-	-
	**Australasian**	3.7223	1.0285	3.6190	0.0004
	Indo-Malayan	0.5942	0.6546	0.9077	0.3650
	Nearctic	0.2624	0.7325	0.3582	0.7205
	Neotropical	0.4112	0.4583	0.8971	0.3706
	Palearctic	0.2143	1.2444	0.1722	0.8634
	Spatial autocorrelation				
	Range	0.0004			
	Nugget	0.1076			
	Random effect (SD)				
	Intercept	1.4468			
	Residual	1.1676			
SES_{mntd}	Intercept	-2.9154	0.1171	-24.8902	< 0.0001
	**Latitude**	0.4127	0.1124	3.6709	0.0003
	Latitude^2	0.0957	0.0961	0.9965	0.3202
	Size	0.1369	0.0917	1.4920	0.1372
	Spatial autocorrelation				
	Range	0.0004			
	Nugget	0.0387			
	Random effect (SD)				
	Intercept	1.0970			
	Residual	1.2451			

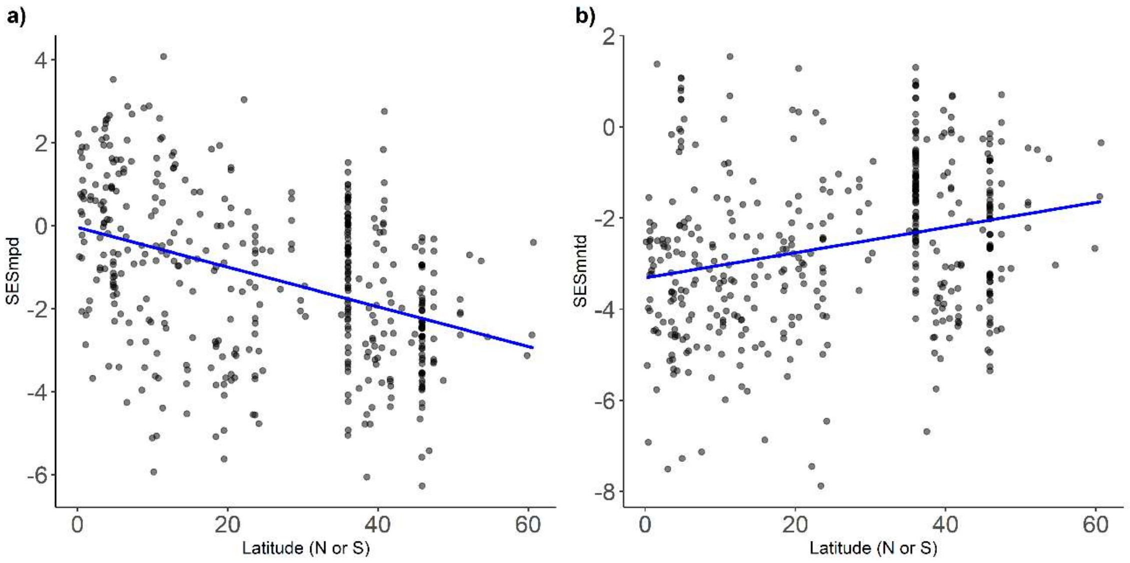

Figure S3. Effect of latitude on phylogenetic diversity of woody communities without gymnosperm species. **(a)** Standardised effect size of mean pairwise distance (SES_{mpd}). **(b)** Standardised effect size of mean nearest taxon distance (SES_{mntd}). Multiple data points may be superimposed. Lines are based on the predicted values from the top-ranked models (Supplementary Table S3).

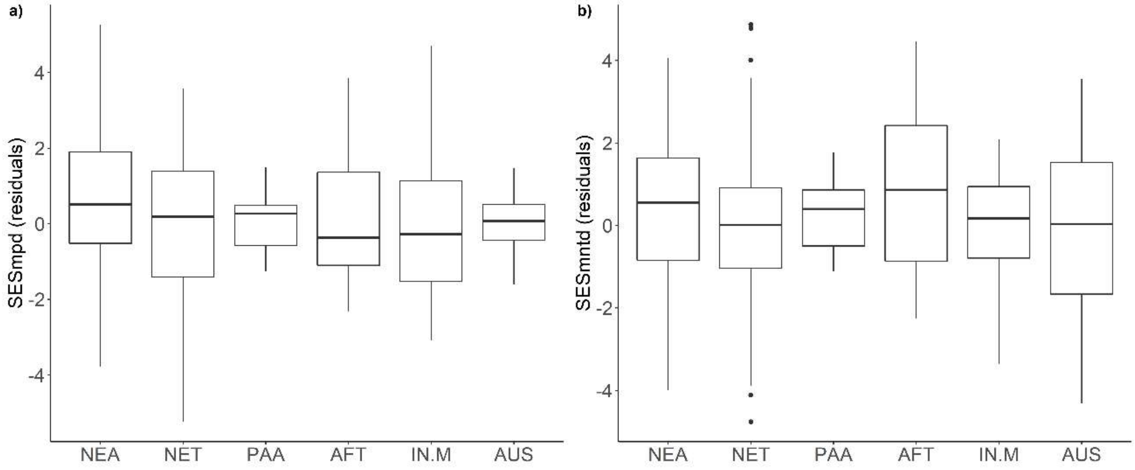

Figure S4. Partial residuals plots for biogeographic realms on phylogenetic diversity of woody communities without gymnosperm species in the top-ranked models while holding all other fixed effects constant (see Table S4 for parameter estimates). **(a)** Standardised effect size of the mean pairwise distance (SES_{mpd}), **(b)** Standardised effect size of the mean nearest taxon distance (SES_{mntd}). NEA. = Nearctic (N = 206); NET = Neotropical (N = 175); PAA = Palearctic (N = 11); AFT = Afrotropical (N = 32); IN.M = Indo.Malayan (N = 16); AUS = Australasian (N = 4). Horizontal lines inside the boxes = median values, boxes = 25% and 75% quartiles, vertical lines = 10% and 90% percentiles, dots = outliers. Horizontal lines inside the boxes = median values, boxes = 25% and 75% quartiles, vertical lines = 10% and 90% percentiles, dots = outliers. Note that biogeographic realms are not included in the top-ranked model with SES_{mntd} as response variable.

Table S5. Relationships between latitude and mean annual temperature, latitude and annual precipitaton, and mean annual temperature and annual precipitation in woody and herbaceous plant communities, using Pearson's product-moment correlation.

Community type	latitude *vs.* temperature		latitude *vs.* precipitation		temperature *vs.* precipitation	
	r	*p*	*r*	*p*	*r*	*p*
Woody	-0.74	<0.0001	-0.46	<0.0001	0.58	<0.0001
Herbaceous	-0.60	<0.0001	-0.51	<0.0001	0.60	<0.0001

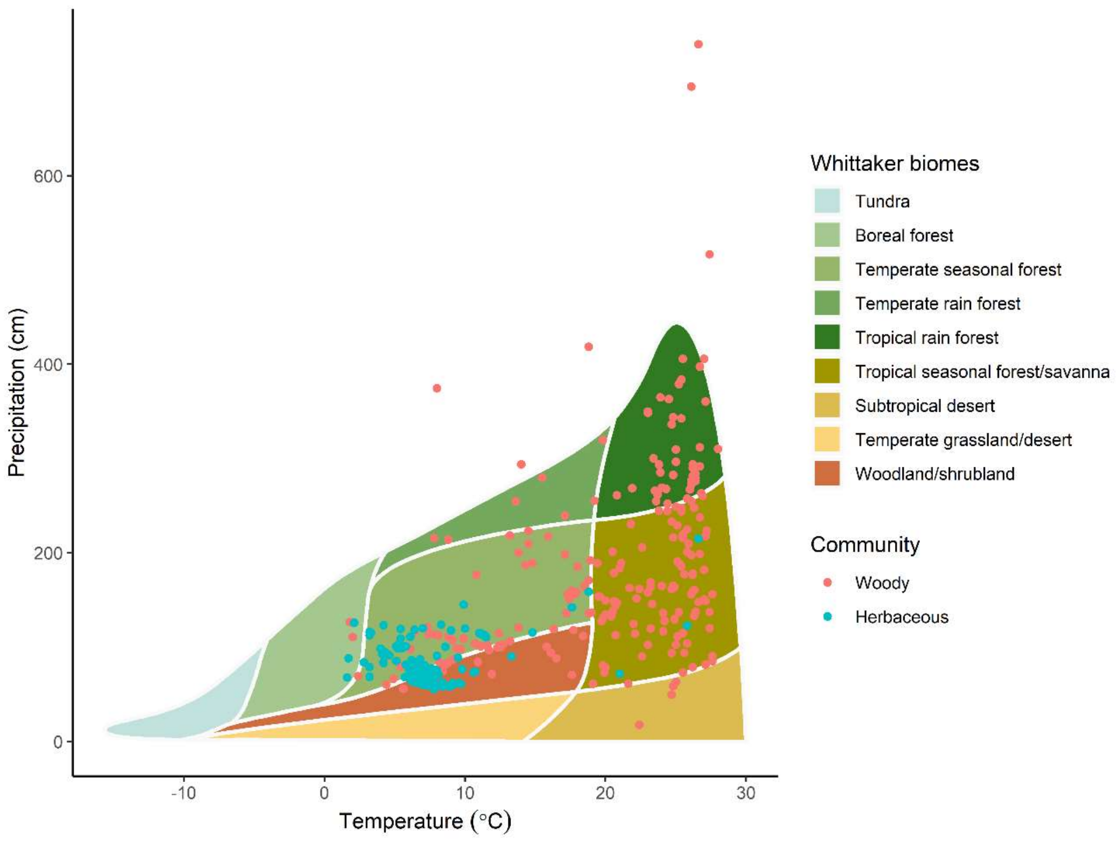

Figure S5. Distribution of woody and herbaceous communities in climate space using a Whittaker plot.

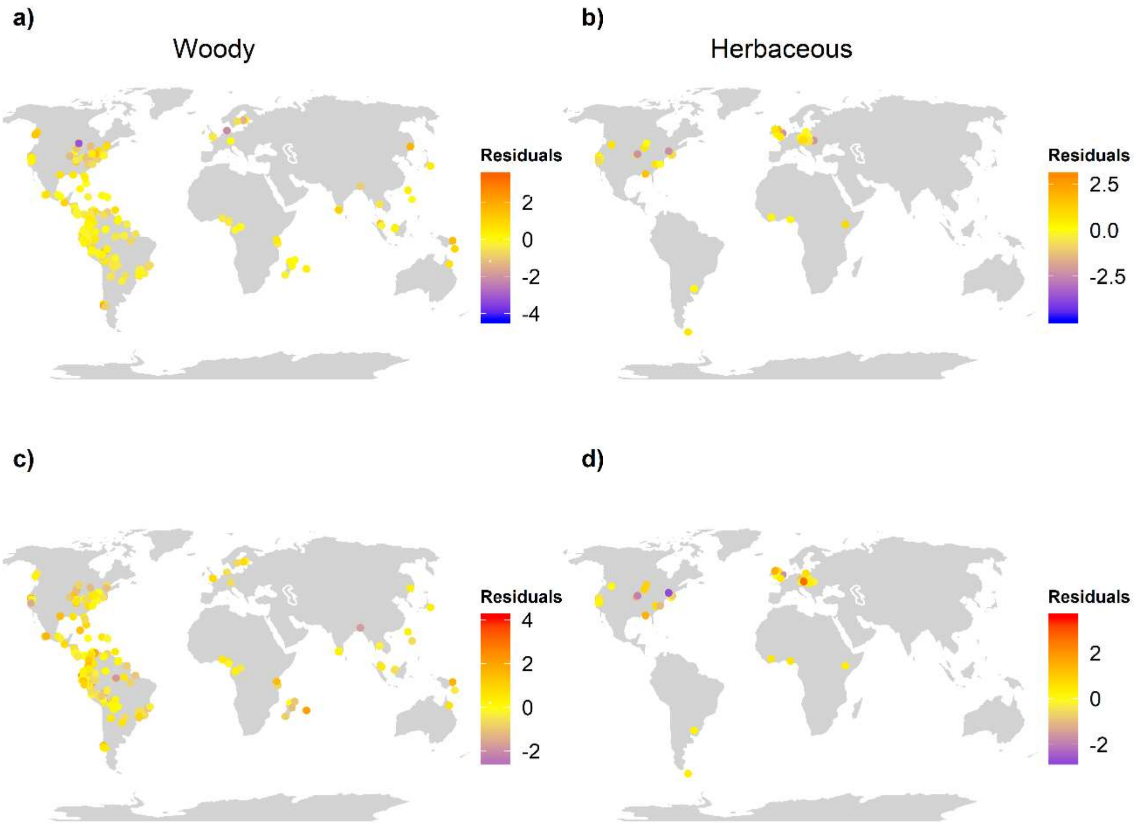

Figure S6. Spatial patterns of normalised residuals in the top-ranked models on community phylogenetic diversity. **(a, b)** = standardised effect size of the mean pairwise distance (SES_{mpd}). **(c, d)** = standardised effect size of the mean nearest taxon distance (SES_{mntd}). Study identification was included as a random effect variable in all models.

2. References for the supplementary results

1. Paradis, E. & Schliep, K. ape 5.0: an environment for modern phylogenetics and evolutionary analyses in R. *Bioinformatics* **35,** 526–528 (2019).

2. Kembel, S. W. *et al.* Picante: R tools for integrating phylogenies and ecology. *Bioinformatics* **26,** 1463–1464 (2010).

3. Qian, H., Zhang, Y., Zhang, J. & Wang, X. Latitudinal gradients in phylogenetic relatedness of angiosperm trees in North America. *Glob. Ecol. Biogeogr.* **22,** 1183–1191 (2013).

4. Kerkhoff, A. J., Moriarty, P. E. & Weiser, M. D. The latitudinal species richness gradient in New World woody angiosperms is consistent with the tropical conservatism hypothesis. *Proc. Natl. Acad. Sci.* **111,** 8125–8130 (2014).

5. Qian, H., Jin, Y. & Ricklefs, R. E. Phylogenetic diversity anomaly in angiosperms between eastern Asia and eastern North America. *Proc. Natl. Acad. Sci.* **114**, 11452–11457 (2017).

6. Kubota, Y., Kusumoto, B., Shiono, T. & Ulrich, W. Environmental filters shaping angiosperm tree assembly along climatic and geographic gradients. *J. Veg. Sci.* **29,** 607–618 (2018).

7. Wiens, J. J. *et al.* Niche conservatism as an emerging principle in ecology and conservation biology. *Ecol. Lett.* **13,** 1310–1324 (2010).

8. Bond, W. J. The tortoise and the hare: ecology of angiosperm dominance and gymnosperm persistence. *Biol. J. Linn. Soc.* **36,** 227–249 (1989).

3. Supplementary References. Literature used to compile the data for this study.

1. Williams, A. B. the composition and dynamics of a beech-maple climax community. *Ecol. Monogr.* **6,** 317–408 (1936).

2. Weaver, J. E. & Bruner, W. E. A seven-year quantitative study of succession in grassland. *Ecol. Monogr.* **15,** 297–319 (1945).

3. Weaver, J. E. & Albertson, F. W. Resurvey of grasses, forbs, and underground plant parts at the end of the great drought. *Ecol. Monogr.* **13,** 63–117 (1943).

4. Vevers, H. G. The land vegetation of Ailsa Craig. *J. Ecol.* **24,** 424 (1936).

5. Weaver, J. E. & Fitzpatrick, T. J. The prairie. *Ecol. Monogh., 4*, 109–295 (1934).

6. Tansley, A. G. Studies of the vegetation of the English Chalk: ii. Early stages of redevelopment of woody vegetation on chalk grassland. *J. Ecol.* **10,** 168 (1922).

7. Summerhayes, V. S. The effect of voles (*Microtus agrestis*) on vegetation. *J. Ecol.* **29,** 14 (1941).

8. Stallard, H. Secondary succession in the climax forest formations of Northern Minnesota. *Ecology* **10,** 476–547 (1929).

9. Sarker, S. K., Sonet, S. S., Mohasinul Haque, M. & Sharmin, M. Disentangling the role of soil in structuring tropical tree communities at Tarap Hill Reserve of Bangladesh. *Ecol. Res.* **28,** 553–565 (2013).

10. Potzger, J. E. The vegetation of Mackinac Island, Michigan: An ecological eurvey. *Am. Midl. Nat.* **25,** 298 (1941).

11. Porembski, S., Brown, G. & Barthlott, W. An inverted latitudinal gradient of plant diversity in shallow depressions on Ivorian inselbergs. *Vegetatio* **117,** 151–163 (1995).

12. Pitt-Schenkel, C. J. W. Some important communities of warm temperate rain forest at magamba, West Usambara, Tanganyika Territory. *J. Ecol.* **26,** 50 (1938).

13. Pinto, M. F. *et al.* Floristic and vegetation structure of a grassland plant community on shallow basalt in Southern Brazil. *Acta Bot. Brasilica* **27,** 162–179 (2013).

14. Pessin, L. J. The effect of vegetation on the growth of longleaf pine seedlings. *Ecol. Monogr.* **8,** 115–149 (1938).

15. Oosting, H. J. An ecological analysis of the plant communities of Piedmont, North Carolina. *Am. Midl. Nat.* **28,** 1 (1942).

16. Olmsted, C. E. Vegetation of certain sand plains of Connecticut. *Bot. Gaz.* **99,** 209–300 (1937).

17. Oba, G., Vetaas, O. R. & Stenseth, N. C. Relationships between biomass and plant species richness in arid-zone grazing lands. *J. Appl. Ecol.* **38,** 836–845 (2001).

18. Mulvania, M. Ecological survey of a Florida scrub. *Ecology* **12,** 528–540 (1931).

19. Merunková, K. & Chytrý, M. Environmental control of species richness and composition in upland grasslands of the southern Czech Republic. *Plant Ecol.* **213,** 591–602 (2012).

20. Mark, A. F., Dickinson, K. J. M., Allen, J., Smith, R. & West, C. J. Vegetation patterns, plant distribution and life forms across the alpine zone in southern Tierra del Fuego, Argentina. *Austral Ecol.* **26,** 423–440 (2001).

21. Lutz, H. J. The vegetation of Heart's Content, a virgin forest in Northwestern Pennsylvania. *Ecology* **11,** 1–29 (1930).

22. E. W. Jones. The structure and reproduction of the virgin forest of the north temperate zone. *New Phytol.* **44,** 130–148 (1945).

23. Hough, A. F. A Climax forest community on East Tionesta Creek in Northwestern Pennsylvania. *Ecology* **17,** 9–28 (1936).

24. Hope-Simpson, J. F. Studies of the vegetation of the English Chalk: VIII. A second survey of the chalk grasslands of the South Downs. *J. Ecol.* **29,** 217 (1941).

25. Hall, T. F. & Penfound, W. T. A phytosociological study of a cypress-gum swamp in Southeastern Louisiana. *Am. Midl. Nat.* **21,** 378 (1939).

26. Giaretta, A., Menezes, L. F. T. de & Pereira, O. J. Structure and floristic pattern of a coastal dunes in southeastern Brazil. *Acta Bot. Brasilica* **27,** 87–107 (2013).

27. Franco, G. A. D. C. *et al.* Importância dos remanescentes florestais de Embu (SP, Brasil) para a conservação da flora regional. *Biota Neotrop.* **7,** 145–161 (2007).

28. Davis, T. A. W. & Richards, P. W. The vegetation of Moraballi Creek, British Guiana: An ecological study of a limited area of tropical rain forest. Part I. *J. Ecol.* **21,** 350 (1933).

29. Daubenmire, R. F. An ecological study of the vegetation of Southeastern Washington and adjacent Idaho. *Ecol. Monogr.* **12,** 53–79 (1942).

30. Daubenmire, R. F. The “big woods” of Minnesota: Its structure, and relation to climate, fire, and soils. *Ecol. Monogr.* **6,** 233–268 (1936).

31. Conard, H. S. The plant associations of Central Long Island. a study in descriptive plant sociology. *Am. Midl. Nat.* **16,** 433 (1935).

32. Chytrý, M. *et al.* The most species-rich plant communities in the Czech Republic and Slovakia (with new world records). *Preslia* **87,** 217–278 (2015).

33. Chapman, V. J. Studies in salt-marsh ecology sections IV and V. *J. Ecol.* **27,** 160 (1939).

34. Cain, S. A. & Penfound, W. T. *Aceretum rubri*: The red maple swamp forest of Central Long Island. *Am. Midl. Nat.* **19,** 390 (1938).

35. Brown, D. M. Vegetation of Roan Mountain: A phytosociological and successional study. *Ecol. Monogr.* **11,** 61–97 (1941).

36. Billings, W. D. The structure and development of old field shortleaf pine stands and certain associated physical properties of the soil. *Ecol. Monogr.* **8,** 437–500 (1938).

37. Young, V. A. Certain sociological aspects associated with plant competition between native and foreign species in a saline area. *Ecology* ***17*,** 133-142 (1936).

38. Aikman, J. M. & Smelser, A. W. The structure and environment of forest communities in Central Iowa. *Ecology* **19,** 141–150 (1938).

39. Beard, J. S. The natural vegetation of the Island of Tobago, British West Indies. *Ecol. Soc. Am.* **14,** 135–163 (1944).

40. Balátová-Tuláčková, E. Feuchtwiesen- und hochstaudengesellschaften des landschaftsschutzgebietes lužické hory und der angrenzenden randgebiete (Nordböhmen). *Verh. Zool.-Bot. Ges. Österreich* **134,** 233–304 (1997).

41. Carvalho, D. A. & Martins, F. R. Shrub and tree species composition in the cerrados of Southwest Minas Gerais. (A flora arbóreo-arbustiva dos cerrados do sudoeste de Minas Geras). *Cerne* **15,** 142–154. (2009).

42. Jones, E. W. Ecological studies on the rain forest of Southern Nigeria: IV (continued). The plateau forest of the Okomu Forest Reserve. *J. Ecol.* **44,** 83–117 (1956).

43. Kłys, H., & Naturalna, J. Assessing the impact of European beaver (Castor fiber) on the organisation of plant communities, a case study from the lower reaches of the River Liswarta. *Opol. Sci. Soc. Nat. J.* **41,** 79–100 (2008).

44. Martincová, J., & Ondrášek, L. Grassland monitoring of meadows in the region around Banská Bystrica. *Czech J. Gen. and Plant Breed.* **46,** S40–S44 (2010).

45. Oosting, H. J. & Billings, W. D. The red fir forest of the Sierra Nevada: Abietum magnificae. *Ecol. Monogr.* **13,** 259–274 (1943).

46. Prajs, B., & Antkowiak, W. Grassland ecosystems in the varied hydrological and ecological conditions of the Kulawa River valley. *Pol. J. Environ. Stud.* **19,** 131–139 (2010).

47. Tansley, A. G., & Adamson, R. S. Studies of the vegetation of the English Chalk: III. The chalk grasslands of Hampshire-Sussex border. *J. Ecol.* **13,** 177–223 (1925).

48. Vaughan, R. E., & Wiehe, P. O. Studies on the vegetation of Mauritius: I. A preliminary survey of the plant communities. *J. Ecol.* **25,** 289–343 (1937).

49. Watt, A. S. The beech associes. *J. Ecol.* **12,** 181–193 (1924).

50. The Alwyn H. Gentry forest transect dataset. Retrieved from http://salvias.net/pages/database_info.php
