# Supplementary material for: Contrasting latitudinal patterns in phylogenetic diversity between woody and herbaceous communities
Source: Sci Rep. 2019 Apr 23;9:6443. doi: 10.1038/s41598-019-42827-1 (PMC6478853; doi:10.1038/s41598-019-42827-1)
Supplement: Supplementary file 3 — R Scripts [file 41598_2019_42827_MOESM3_ESM.pdf]

```
#################################################################
                              #
#
                              #                            R script
#
                              #
#

#################################################################



# Contrasting latitudinal patterns in phylogenetic diversity between woody
and herbaceous communities
```


```
# Jhonny C Massante*(1), Lars Götzenberger(2), Krista Takkis(3), Tiit
Hallikma(3),
# Ants Kaasik(1), Lauri Laanisto(3), Michael J Hutchings(4) and Pille
Gerhold(1)

#(1) Institute of Ecology and Earth Sciences, University of Tartu, Tartu
50410, Estonia
#(2) Institute of Botany, Academy of Sciences of the Czech Republic, CZ-
37982 Trebon, Czech Republic
#(3) Institute of Agricultural and Environmental Sciences, Estonian
University of Life Sciences, Tartu 51014, Estonia
#(4) School of Life Sciences, University of Sussex, Falmer, Brighton,
Sussex BN1 9QG, UK

#(*) Corresponding author: Institute of Ecology and Earth Sciences,
University of Tartu, Tartu 51014, Estonia. Phone: +372 737 5853; E-mail:
jhonny.massante@ut.ee.
```


```
# This section of the supplementary information contains R scripts used to
standardise species names according to
# The Plant List, to produce the phylogenetic tree and phylogenetic
indexes, and all statistical analyses
# as well as sources of specific codes used to derive climatic data from
WorldClim database and global map using Robinson projection.



# Load required packages

library(Taxonstand)# Standardisation of species names according to The
Plant List (version 1.1)
library(ape)# Phylogenetic analysis
library(phytools)# Phylogenetic analysis
library(picante)# Calculation phylogenetic diversity
library(nlme)# Mixed-effect models
library(tidyverse)# Data manipulation and plots
library(ggpubr)# Group ggplots
library(ggformula)# Show lines in the figure 2 and supplementary figure S3.
library(MuMIn)# Model selection and marginal/conditional r square
library(maps)# Plot global maps with woody and herbaceous communities and
normalised residuals
library(ggmap)# Plot global maps with woody and herbaceous communities and
normalised residuals
library(rasterVis)# Plot global maps with woody and herbaceous communities
and normalised residuals
library(raster)# Plot global maps with woody and herbaceous communities and
normalised residuals
library(rworldmap)# Plot global maps with woody and herbaceous communities
and normalised residuals
library(rworldxtra)# Plot global maps with woody and herbaceous communities
and normalised residuals
library(rgdal)# Plot global maps with woody and herbaceous communities and
normalised residuals
library(maptools)# Plot global maps with woody and herbaceous communities
and normalised residuals
library(plotbiomes)# Plot Whittaker biomes on climate space



# Set working directory
getwd()
setwd("working.directory")



#####################################################################
############   CLIMATIC DATA FROM WORLDCLIM DATABASE   ############
#####################################################################

#We extracted data from the database WorldClim using the
#script available at https://gist.github.com/kgturner/6643334




#####################################################################
############       SPECIES NAMES STANDARDISATION       ############
#####################################################################

# The"species.names" file must be a data frame with five columns as
described by the
```

```
# authors of the package in its documentation.
# It returns a new data frame with full species names standardised
according to The Plant List, their genus and families.
# For those taxa identified to genus level or with mispelled names, we
standardised their names manually
# according to The Plant List
species.names <- read.csv("species.names.csv", header = TRUE, sep = ";")

# Species names in full
names.corrected <- TPL(species.names$Full.name, corr=TRUE)

# Export to a table separated by comma
write.table(names.corrected, "names.corrected.csv", sep=",")




##############################################################################
#
#########     PHYLOGENETIC TREE AND PHYLOGENETIC DIVERSITY INDEXES
##########
##############################################################################
#

# Function used to add missing taxa to the phylogeny using scenario 3
described in the main text and in the reference 65
source(S.PhyloMaker)# Available from the reference 65


### Phylogenetic tree ###

# Load species list. It must be a data frame with three columns as
specified in the reference 65
splist<-read.csv("species.list.csv", header = T, sep = ";")

# Load nodes file
nodes<-read.csv("nodes.csv", header = T, sep = ";")# Available from the
reference 65

# Load megaphylogeny
gbotb <- read.tree(file = "GBOTB.tre")# Available from the reference 63
tree <- gbotb#rename the phylogenetic tree

# Create phylogenetic tree and adds missing taxa to it using scenario 3
described in the main text and in the reference 65
result<-S.PhyloMaker(splist, tree, nodes, output.splist = T, scenarios =
"S3")


# Extract the phylogeny from the result
S3<-result$Scenario.3



####   Prepare data to run SES indexes for woody communities  ####

# Load woody community file. It must be a matrix with species names in
columns and site names in rows
# We used a presence/absence matrix in our analyses
comm.woody<-read.csv("comm.woody.csv", header = T, row.names = 1, sep =
";",
                     check.names = FALSE)

# Check dimensions (number of rows and columns)
dim(comm.woody)

# Replace any space by "_"
colnames(comm.woody) <- gsub(" ", "_", colnames(comm.woody))

# Pruning the tree with woody species only
S3.woody.pruned<- prune.sample(comm.woody, S3)

# Sort the columns of "comm" to be in the same order as the tip labels of
the phylogeny
comm.woody<- comm.woody[, S3.woody.pruned$tip.label]

# Create species distance matrix from the pruned tree
dist3.woody<-cophenetic(S3.woody.pruned)

# Dimensions (number of rows and columns) of matrix and community data
dim(dist3.woody)
dim(comm.woody)

# Calculate standardised effect size of mean pairwise distance (SESmpd)
sesmpd.woody<- ses.mpd(as.matrix(comm.woody), (dist3.woody), null.model =
"taxa.labels", runs = 999)
write.table(sesmpd.woody, "sesmpd.woody.csv", sep=",")


# Calculate standardised effect size of mean nearest taxon distance
(SESmntd)
sesmntd.woody<- ses.mntd(as.matrix(comm.woody), (dist3.woody), null.model =
"taxa.labels", runs = 999)
write.table(sesmntd.woody, "sesmntd.woody.csv", sep=",")




####   Prepare data to run SES indexes for herbaceous communities  ####
```

```
# Load herbaceous community file. It must be a matrix with species names in
columns and site names in rows
# We used a presence/absence matrix in our analyses
comm.herbs<-read.csv("comm.herb.csv", header = T, row.names = 1, sep = ";",
                     check.names = FALSE)

#Chech dimensions (number of rows and columns)
dim(comm.herbs)

# Replace any space by "_"
colnames(comm.herbs) <- gsub(" ", "_", colnames(comm.herbs))

# Pruning the tree with herbaceous species only
S3.herbs.pruned<- prune.sample(comm.herbs, S3)

# Sort the columns of "comm" to be in the same order as the tip labels of
the phylogeny
comm.herbs<- comm.herbs[, S3.herbs.pruned$tip.label]

# Create species distance matrix from the pruned tree
dist3.herbs<-cophenetic(S3.herbs.pruned)

# Dimensions (number of rows and columns) of matrix and community data
dim(dist3.herbs)
dim(comm.herbs)

# Calculate standardised effect size of mean pairwise distance (SESmpd)
sesmpd.herbs<- ses.mpd(as.matrix(comm.herbs), (dist3.herbs), null.model =
"taxa.labels", runs = 999)
write.table(sesmpd.herbs, "sesmpd.herbs.csv", sep=",")


# Calculate standardised effect size of mean nearest taxon distance
(SESmntd)
sesmntd.herbs<- ses.mntd(as.matrix(comm.herbs), (dist3.herbs), null.model =
"taxa.labels", runs = 999)
write.table(sesmntd.herbs, "sesmntd.herbs.csv", sep=",")



###############################################################################################
#
################               STATISTICAL ANALYSES AND FIGURES
####################
###############################################################################################
#



# Load the supplementary dataset.  It is available as a excel file
separated by comma
# Depending on the system, it can be loaded with the argument sep = "," or
sep = ";" as shown below
data <- read.csv("Supplementary.Dataset.csv", header = T, sep = ";")

# Split the data into woody and herbaceous communities
# Woody coomunities
woody <- data %>%
  filter(community.type == "Woody")

# Herbaceous communities
herb <- data %>%
  filter(community.type != "Woody")


#=====================================================================================================================
=

### Grouping the data to be analyzed with lme function from the lmer
package ###
group.woody.mpd <- groupedData(ses.mpd ~ latitude|no.article, data = woody)
group.woody.mntd <- groupedData(ses.mntd ~ latitude|no.article, data =
woody)

group.herb.mpd <- groupedData(ses.mpd ~ latitude|no.article, data = herb)
group.herb.mntd <- groupedData(ses.mntd ~ latitude|no.article, data = herb)

#=====================================================================================================================
=

### Base for global maps ###

# Read shapefile downloaded from https://www.naturalearthdata.com/
wmap <- readOGR(dsn="ne_110m_land", layer="ne_110m_land")

# Convert to dataframe
wmap_df <- fortify(wmap)

# ggplot theme
theme_opts <- list(theme(panel.grid.minor = element_blank(),
                         panel.grid.major = element_blank(),
                         panel.background = element_blank(),
                         plot.background = element_rect(fill="white"),
                         panel.border = element_blank(),
                         axis.line = element_blank(),
                         axis.text.x = element_blank(),
                         axis.text.y = element_blank(),
                         axis.ticks = element_blank(),
```

```
                          axis.title.x = element_blank(),
                          axis.title.y = element_blank(),
                          plot.title = element_text(size=15),
                          legend.text = element_text(size=12),
                          legend.title = element_text(size = 12),
                          plot.margin = unit(c(-225, 20, -225, -65), "pt"),
                          legend.justification = c(0,1),
                          legend.position = c(0.9, 0.8)))




# Reproject from longlat to robinson
wmap_robin <- spTransform(wmap, CRS("+proj=robin"))
wmap_df_robin <- fortify(wmap_robin)


# Ordering biogeographic realms' and vegetation types' levels to prevent
alphabetic order

# Woody
woody$realm <- factor(woody$realm, levels = c("Nearctic", "Neotropical",
"Palearctic", "Afrotropical", "Indo.Malayan", "Australasian"), ordered = F)
woody$new.veg.type <- factor(woody$new.veg.type, levels = c("closed",
"semi-open", "open"), ordered = F)

# Herbaceous
herb$realm <- factor(herb$realm, levels = c("Nearctic", "Neotropical",
"Palearctic", "Afrotropical"), ordered = F)
herb$new.veg.type <- factor(herb$new.veg.type, levels = c("closed",
"open"), ordered = F)



#==================================================================================================================================
=

### Models with SESmpd as response variable in woody communities ###

model.1.w.mpd <- lme(ses.mpd ~ scale(poly(abs.lat, 2)) +
scale(plot.size.ha) + realm,
                     data = group.woody.mpd, random = ~1|no.article, method
= "ML",
                     correlation = corExp(form = ~longitude +
latitude|no.article, nugget = T),
                     na.action = na.exclude)

model.2.w.mpd <- lme(ses.mpd ~ scale(poly(abs.lat, 2)) +
scale(plot.size.ha) + new.veg.type,
                     data = group.woody.mpd, random = ~1|no.article, method
= "ML",
                     correlation = corExp(form = ~longitude +
latitude|no.article, nugget = T),
                     na.action = na.exclude)

model.3.w.mpd <- lme(ses.mpd ~ scale(poly(abs.lat, 2)) +
scale(plot.size.ha) + realm + new.veg.type,
                     data = group.woody.mpd, random = ~1|no.article, method
= "ML",
                     correlation = corExp(form = ~longitude +
latitude|no.article, nugget = T),
                     na.action = na.exclude)

model.4.w.mpd <- lme(ses.mpd ~ scale(poly(abs.lat, 2)) +
scale(plot.size.ha),
                     data = group.woody.mpd, random = ~1|no.article, method
= "ML",
                     correlation = corExp(form = ~longitude +
latitude|no.article, nugget = T),
                     na.action = na.exclude)


# Model selection based on AIC values using MuMIn package
model.sel(model.1.w.mpd, model.2.w.mpd, model.3.w.mpd, model.4.w.mpd)

# Get marginal and condicional R square using MuMIn package
r.squaredGLMM(model.2.w.mpd)
r.squaredGLMM(model.1.w.mpd)
r.squaredGLMM(model.4.w.mpd)
r.squaredGLMM(model.3.w.mpd)



# Refit top-ranked model with REML
model.2.w.mpd.reml <- update(model.2.w.mpd, method = "REML")

# Get anova table from the top-ranked model fitted with REML using marginal
sum of squares
anova.lme(model.2.w.mpd.reml, type = 'marginal')

#Get parameter estimates from the top-ranked model fitted with REML
summary(model.2.w.mpd.reml)

# Get predicted values from the top-ranked model to plot the line
woody$pred.mpd.w <- predict(model.2.w.mpd.reml)

# Get normalised residuals from the top-ranked model
resid.model.2.w.mpd.reml <- residuals(model.2.w.mpd.reml, type =
"normalized")
```

```
# Plot SESmpd along latitudinal gradient
woody.mpd.lat <- ggplot(woody, aes(abs.lat, ses.mpd)) +
  scale_y_continuous(name="SESmpd", breaks=pretty(woody$ses.mpd, n = 6))+
  geom_point(alpha = 0.5) +
  theme_classic()+
  scale_x_continuous(name="")+
  geom_lm(mapping = NULL, formula = y ~ poly(x, 2), size = 1, colour =
"blue")+
  ggtitle(label = "Woody")+
  theme(plot.title = element_text(hjust = 0.5))+
  theme(axis.title.y = element_text(size = 15))

# Create a data frame with normalised residuals to be plotted on global map
top.woody.sesmpd <- project(cbind(woody$longitude, woody$latitude),
proj="+init=ESRI:54030")
top.woody.sesmpd <- as.data.frame(top.woody.sesmpd)
top.woody.sesmpd <- cbind(top.woody.sesmpd, resid.model.2.w.mpd.reml)
names(top.woody.sesmpd) <- c("LONGITUDE", "LATITUDE", "Residuals")

# Midpoint in the color scale (also used in the following global maps of
normalised residuals)
mid = 0

# Plot normalised residuals on global map with ggplot theme created in the
lines 232-245
  woody.mpd <- ggplot(wmap_df_robin, aes(long,lat, group=group)) +
    geom_polygon(colour="white", fill="light gray") +
    ggtitle("Woody") +
    theme(plot.title = element_text(hjust = 0.5))+
    coord_equal() +
    theme_opts+
    geom_point(data = top.woody.sesmpd, aes(LONGITUDE, LATITUDE, color =
Residuals, group = NULL, fill = NULL))+
    scale_color_gradient2(midpoint = mid, low="blue", mid = "yellow",
high="red", space = "Lab")+
    theme(legend.title = element_text(size=10, face="bold"))


#==============================================================================================================================
=

### Models with SESmntd as response variable in woody communities ###

model.1.w.mntd <- lme(ses.mntd ~ scale(poly(abs.lat, 2)) +
scale(plot.size.ha) + realm,
                      data = group.woody.mntd, random = ~1|no.article,
method = "ML",
                      correlation = corExp(form = ~longitude +
latitude|no.article, nugget = T),
                      na.action = na.exclude)

model.2.w.mntd <- lme(ses.mntd ~ scale(poly(abs.lat, 2)) +
scale(plot.size.ha) + new.veg.type,
                      data = group.woody.mntd, random = ~1|no.article,
method = "ML",
                      correlation = corExp(form = ~longitude +
latitude|no.article, nugget = T),
                      na.action = na.exclude)

model.3.w.mntd <- lme(ses.mntd ~ scale(poly(abs.lat, 2)) +
scale(plot.size.ha) + realm + new.veg.type,
                      data = group.woody.mntd, random = ~1|no.article,
method = "ML",
                      correlation = corExp(form = ~longitude +
latitude|no.article, nugget = T),
                      na.action = na.exclude)

model.4.w.mntd <- lme(ses.mntd ~ scale(poly(abs.lat, 2)) +
scale(plot.size.ha),
                      data = group.woody.mntd, random = ~1|no.article,
method = "ML",
                      correlation = corExp(form = ~longitude +
latitude|no.article, nugget = T),
                      na.action = na.exclude)


# Model selection based on AIC values using MuMIn package
model.sel(model.1.w.mntd, model.2.w.mntd, model.3.w.mntd, model.4.w.mntd)

# Get marginal and condicional R square using MuMIn package
r.squaredGLMM(model.4.w.mntd)
r.squaredGLMM(model.2.w.mntd)
r.squaredGLMM(model.1.w.mntd)
r.squaredGLMM(model.3.w.mntd)


# Refit top-ranked model with REML
model.4.w.mntd.reml <- update(model.4.w.mntd, method = "REML")

# Get anova table from the top-ranked model fitted with REML
anova.lme(model.4.w.mntd.reml, type = 'marginal')

# Get parameter estimates from the top-ranked model fitted with REML
summary(model.4.w.mntd.reml)

# Get predicted values from the top-ranked model to plot the line
woody$pred.mntd.w <- predict(model.4.w.mntd.reml)

# Get normalised residuals from the top-ranked model
```

```
resid.model.4.w.mntd.reml <- residuals(model.4.w.mntd.reml, type =
"normalized")


# Plot SESmntd along latitudinal gradient
woody.mntd.lat <- ggplot(woody, aes(abs.lat, ses.mntd)) +
  scale_y_continuous(name="SESmntd", breaks=pretty(woody$ses.mntd, n = 6))+
  geom_point(alpha = 0.5) +
  theme_classic()+
  scale_x_continuous(name="Latitude (N or S)")+
  geom_lm(mapping = NULL, formula = y ~ x, size = 1, colour = "blue")+
  ggtitle(label = "")+
  theme(plot.title = element_text(hjust = 0.5))+
  theme(axis.title.y = element_text(size = 15))+
  theme(axis.title.x = element_text(size = 15))

# Create a data frame with normalised residuals to be plotted on global map
top.woody.sesmntd <- project(cbind(woody$longitude, woody$latitude),
proj="+init=ESRI:54030")
top.woody.sesmntd <- as.data.frame(top.woody.sesmntd)
top.woody.sesmntd <- cbind(top.woody.sesmntd, resid.model.4.w.mntd.reml)
names(top.woody.sesmntd) <- c("LONGITUDE", "LATITUDE", "Residuals")


# Plot normalised residuals on global map with ggplot theme created in the
lines 232-245
woody.mntd <- ggplot(wmap_df_robin, aes(long,lat, group=group)) +
  geom_polygon(colour="white", fill="light gray") +
  ggtitle("") +
  theme(plot.title = element_text(hjust = 0.5))+
  coord_equal() +
  theme_opts+
  geom_point(data = top.woody.sesmntd, aes(LONGITUDE, LATITUDE, color =
Residuals, group = NULL, fill = NULL))+
  scale_color_gradient2(midpoint = mid, low="blue", mid = "yellow",
high="red", space = "Lab")+
  theme(legend.title = element_text(size=10, face="bold"))


#==================================================================================================================================
=

### Models with SESmpd as response variable in herbaceous communities ###

model.1.h.mpd <- lme(ses.mpd ~ scale(poly(abs.lat, 2)) +
scale(plot.size.ha) + realm,
                     data = group.herb.mpd, random = ~1|no.article, method
= "ML",
                     correlation = corExp(form = ~longitude +
latitude|no.article, nugget = T),
                     na.action = na.exclude)

model.2.h.mpd <- lme(ses.mpd ~ scale(poly(abs.lat, 2)) +
scale(plot.size.ha) + new.veg.type,
                     data = group.herb.mpd, random = ~1|no.article, method
= "ML",
                     correlation = corExp(form = ~longitude +
latitude|no.article, nugget = T),
                     na.action = na.exclude)

model.3.h.mpd <- lme(ses.mpd ~ scale(poly(abs.lat, 2)) +
scale(plot.size.ha) + realm + new.veg.type,
                     data = group.herb.mpd, random = ~1|no.article, method
= "ML",
                     correlation = corExp(form = ~longitude +
latitude|no.article, nugget = T),
                     na.action = na.exclude)

model.4.h.mpd <- lme(ses.mpd ~ scale(poly(abs.lat, 2)) +
scale(plot.size.ha),
                     data = group.herb.mpd, random = ~1|no.article, method
= "ML",
                     correlation = corExp(form = ~longitude +
latitude|no.article, nugget = T),
                     na.action = na.exclude)


# Model selection based on AIC values using MuMIn package
model.sel(model.1.h.mpd, model.2.h.mpd, model.3.h.mpd, model.4.h.mpd)

# Get marginal and condicional R square using MuMIn package
r.squaredGLMM(model.3.h.mpd)
r.squaredGLMM(model.2.h.mpd)
r.squaredGLMM(model.1.h.mpd)
r.squaredGLMM(model.4.h.mpd)


# Refit top-ranked model with REML
model.3.h.mpd.reml <- update(model.3.h.mpd, method = "REML")

# Get anova table from the top-ranked model fitted with REML
anova.lme(model.3.h.mpd.reml, type = 'marginal')

# Get parameter estimates from the top-ranked model fitted with REML
summary(model.3.h.mpd.reml)

# Get predicted values from the top-ranked model to plot the line
herb$pred.mpd.h <- predict(model.3.h.mpd.reml)

# Get normalised residuals from the top-ranked model
```

```
resid.model.3.h.mpd.reml <- residuals(model.3.h.mpd.reml, type =
"normalized")

# Plot SESmpd along latitudinal gradient
herb.mpd.lat <- ggplot(herb, aes(abs.lat, ses.mpd)) +
  scale_y_continuous(name="", breaks=pretty(herb$ses.mpd, n = 6))+
  geom_point(alpha = 0.5) +
  theme_classic()+
  scale_x_continuous(name="")+
  geom_lm(mapping = NULL, formula = y ~ poly(x, 2), size = 1, colour =
"blue")+
  ggtitle(label = "Herbaceous")+
  theme(plot.title = element_text(hjust = 0.5))

# Create a data frame with normalised residuals to be plotted on global map
top.herb.sesmpd <- project(cbind(herb$longitude, herb$latitude),
proj="+init=ESRI:54030")
top.herb.sesmpd <- as.data.frame(top.herb.sesmpd)
top.herb.sesmpd <- cbind(top.herb.sesmpd, resid.model.3.h.mpd.reml)
names(top.herb.sesmpd) <- c("LONGITUDE", "LATITUDE", "Residuals")


# Plot normalised residuals on global map
herb.mpd <- ggplot(wmap_df_robin, aes(long,lat, group=group)) +
  geom_polygon(colour="white", fill="light gray") +
  ggtitle("Herbaceous") +
  theme(plot.title = element_text(hjust = 0.5))+
  coord_equal() +
  theme_opts+
  geom_point(data = top.herb.sesmpd, aes(LONGITUDE, LATITUDE, color =
Residuals, group = NULL, fill = NULL))+
  scale_color_gradient2(midpoint = mid, low="blue", mid = "yellow",
high="red", space = "Lab")+
  theme(legend.title = element_text(size=10, face="bold"))



#==========================================================================================================================
=

### Models with SESmntd as response variable in herbaceous communities ###

model.1.h.mntd <- lme(ses.mntd ~ scale(poly(abs.lat, 2)) +
scale(plot.size.ha),
                      data = group.herb.mntd, random = ~1|no.article,
method = "ML",
                      correlation = corExp(form = ~longitude +
latitude|no.article, nugget = T),
                      na.action = na.exclude)

model.2.h.mntd <- lme(ses.mntd ~ scale(poly(abs.lat, 2)) +
scale(plot.size.ha) + realm,
                      data = group.herb.mntd, random = ~1|no.article,
method = "ML",
                      correlation = corExp(form = ~longitude +
latitude|no.article, nugget = T),
                      na.action = na.exclude)

model.3.h.mntd <- lme(ses.mntd ~ scale(poly(abs.lat, 2)) +
scale(plot.size.ha) + new.veg.type,
                      data = group.herb.mntd, random = ~1|no.article,
method = "ML",
                      correlation = corExp(form = ~longitude +
latitude|no.article, nugget = T),
                      na.action = na.exclude)

model.4.h.mntd <- lme(ses.mntd ~ scale(poly(abs.lat, 2)) +
scale(plot.size.ha) + realm + new.veg.type,
                      data = group.herb.mntd, random = ~1|no.article,
method = "ML",
                      correlation = corExp(form = ~longitude +
latitude|no.article, nugget = T),
                      na.action = na.exclude)

# Model selection based on AIC values using MuMIn package
model.sel(model.1.h.mntd, model.2.h.mntd, model.3.h.mntd, model.4.h.mntd)

# Get marginal and condicional R square using MuMIn package
r.squaredGLMM(model.4.h.mntd)
r.squaredGLMM(model.3.h.mntd)
r.squaredGLMM(model.2.h.mntd)
r.squaredGLMM(model.1.h.mntd)


# Refit top-ranked model with REML
model.4.h.mntd.reml <- update(model.4.h.mntd, method = "REML")

# Get anova table from the top-ranked model fitted with REML
anova.lme(model.4.h.mntd.reml, type = 'marginal')

#Get parameter estimates from the top-ranked model fitted with REML
summary(model.4.h.mntd.reml)


# Get predicted values from the top-ranked model to plot the line
herb$pred.mntd.h <- predict(model.4.h.mntd.reml)


# Get normalised residuals from the top-ranked model
```

```
resid.model.4.h.mntd.reml <- residuals(model.4.h.mntd.reml, type =
"normalized")

# Plot ses.mpd along latitudinal gradient
herb.mntd.lat <- ggplot(herb, aes(abs.lat, ses.mntd)) +
  scale_y_continuous(name="", breaks=pretty(herb$ses.mntd, n = 6))+
  geom_point(alpha = 0.5) +
  theme_classic()+
  scale_x_continuous(name="Latitude (N or S)")+
  ggtitle(label = "")+
  theme(plot.title = element_text(hjust = 0.5))+
  theme(axis.title.y = element_text(size = 15))+
  theme(axis.title.x = element_text(size = 15))

# Create a data frame with normalised residuals to be plotted on global map
top.herb.sesmntd <- project(cbind(herb$longitude, herb$latitude),
proj="+init=ESRI:54030")
top.herb.sesmntd <- as.data.frame(top.herb.sesmntd)
top.herb.sesmntd <- cbind(top.herb.sesmntd, resid.model.4.h.mntd.reml)
names(top.herb.sesmntd) <- c("LONGITUDE", "LATITUDE", "Residuals")

# Plot normalised residuals on global map
herb.mntd <- ggplot(wmap_df_robin, aes(long,lat, group=group)) +
  geom_polygon(colour="white", fill="light gray") +
  ggtitle("") +
  theme(plot.title = element_text(hjust = 0.5))+
  coord_equal() +
  theme_opts+
  geom_point(data = top.herb.sesmntd, aes(LONGITUDE, LATITUDE, color =
Residuals, group = NULL, fill = NULL))+
  scale_color_gradient2(midpoint = mid, low="blue", mid = "yellow",
high="red", space = "Lab")+
  theme(legend.title = element_text(size=10, face="bold"))




#==================================================================================================================
=

### Plot the global distribution of woody and herbaceous communities ###

# Reordering the levels of community type for a better presentation in the
global map
data$community.type <- factor(data$community.type, levels = c("Woody",
"Herbaceous"))

# Individual communities on the global map
points <- project(cbind(data$longitude, data$latitude),
proj="+init=ESRI:54030")
points <- as.data.frame(points)
points <- cbind(points, data$community.type)
names(points) <- c("LONGITUDE", "LATITUDE", "Community")

# Prepare for high resolution figure
tiff("global.map.plot.tiff", units="in", width=12, height=6, res=600)

# Plot the global map with both woody and herbaceous communities
ggplot(wmap_df_robin, aes(long,lat, group=group)) +
  geom_polygon(colour="white", fill="light gray") +
  labs(title="") +
  coord_equal() +
  theme_opts+
  geom_point(data = points, aes(LONGITUDE, LATITUDE, color = Community,
group = NULL, fill = NULL))

# Clean graphic environment
dev.off()




#==================================================================================================================
=

### Plot all phylogenetic diversity -latitude relationships ###

# Prepare for high resolution figure
tiff("PD.lat.plot.tiff", units="in", width=7, height=6, res=600)

# Plot all figures together
ggarrange(woody.mpd.lat,  herb.mpd.lat, woody.mntd.lat, herb.mntd.lat,
          labels = c("a)", "b)", "c)", "d)"),
          ncol = 2, nrow = 2)
# Clean graphic environment
dev.off()




#==================================================================================================================
=
```

```
### Plot all residuals maps ###

# Prepare for high resolution figure
tiff("residuals.plot.tiff", units="in", width=9, height=7, res=600)

# Plot all residuals of top-ranked models on global map
ggarrange(woody.mpd, herb.mpd, woody.mntd, herb.mntd,
          labels = c("a)", "b)", "c)", "d)"),
          ncol = 2, nrow = 2)
# Clean graphic environment
dev.off()







#===============================================================================================================================
=

# Plot Whittaker biomes

#P repare for high resolution figure
tiff("Whittaker.plot.tiff", units="in", width=8, height=6, res=600)

# Plot Whittaker biomes. Note that temperature (bio1) and precipitation
(bio12) were divided by 10 to be in cm scale
whittaker_base_plot()+
  theme_classic()+
  geom_point(data = data, aes(x = bio1, y = bio12/10, color =
community.type))+
  geom_point(data = data, aes(x = bio1, y = bio12/10, color =
community.type))

# Clean graphic environment
dev.off()





#===============================================================================================================================
=

# Supplementary figures for woody communities (boxplots for realms and
vegetation types)
woody.realm.mpd <- ggplot(woody, aes(realm, model.2.w.mpd.reml$residuals[,
1]))+
  geom_boxplot()+
  theme_classic()+
  scale_y_continuous(name = "SESmpd (residuals)", breaks = c(-6, -3, 0, 3,
6, 9))+
  theme(axis.text.y = element_text(size=17))+
  theme(axis.text.x = element_text(size=17))+
  scale_x_discrete(labels = c("NEA", "NET", "PAA", "AFT", "IN.M", "AUS"),
name = "")+
  theme(axis.title.y = element_text(size = 17))

woody.realm.mntd <- ggplot(woody, aes(realm,
model.4.w.mntd.reml$residuals[, 1]))+
  geom_boxplot()+
  theme_classic()+
  scale_y_continuous(name = "SESmntd (residuals)", breaks = c(-8, -6, -4, -
2, 0, 2, 4))+
  theme(axis.text.y = element_text(size=17))+
  theme(axis.text.x = element_text(size=17))+
  scale_x_discrete(labels = c("NEA", "NET", "PAA", "AFT", "IN.M", "AUS"),
name = "")+
  theme(axis.title.y = element_text(size = 15))

woody.veg.mpd <- ggplot(woody, aes(new.veg.type,
model.2.w.mpd.reml$residuals[, 1]))+
  geom_boxplot()+
  theme_classic()+
  scale_y_continuous(name = "SESmpd (residuals)", breaks = c(-6, -3, 0, 3,
6, 9))+
  theme(axis.text.y = element_text(size=17))+
  theme(axis.text.x = element_text(size=17))+
  scale_x_discrete(labels = c("Closed", "Semi-open", "Open"), name = "")+
  theme(axis.title.y = element_text(size = 17))

woody.veg.mntd <- ggplot(woody, aes(new.veg.type,
model.4.w.mntd.reml$residuals[, 1]))+
  geom_boxplot()+
  theme_classic()+
  scale_y_continuous(name = "SESmntd (residuals)", breaks = c(-8, -6, -4, -
2, 0, 2, 4))+
  theme(axis.text.y = element_text(size=17))+
  theme(axis.text.x = element_text(size=17))+
  scale_x_discrete(labels = c("Closed", "Semi-open", "Open"), name = "")+
  theme(axis.title.y = element_text(size = 17))

# Prepare for high resolution figure
tiff("woody.realm.veg.lat.plot.tiff", units="in", width=14, height=9,
res=600)

# Plot all figures together
ggarrange(woody.realm.mpd, woody.veg.mpd, woody.realm.mntd, woody.veg.mntd,
```

```
          labels = c("a)", "b)", "c)", "d)"),
          ncol = 2, nrow = 2)

# Clean graphic environment
dev.off()





#==============================================================================================================================
=

# Supplementary figures for herb communities (boxplots for realms and
vegetation types)
herb.realm.mpd <- ggplot(herb, aes(realm, model.3.h.mpd.reml$residuals[,
1]))+
  geom_boxplot()+
  theme_classic()+
  scale_y_continuous(name = "SESmpd (residuals)", breaks = c(-8, -6, -4, -
2, 0, 2, 4))+
  theme(axis.text.y = element_text(size=17))+
  theme(axis.text.x = element_text(size=17))+
  scale_x_discrete(labels = c("NEA", "NET", "PAA", "AFT"), name = "")+
  theme(axis.title.y = element_text(size = 17))

herb.realm.mntd <- ggplot(herb, aes(realm, model.4.h.mntd.reml$residuals[,
1]))+
  geom_boxplot()+
  theme_classic()+
  scale_x_discrete(labels = c("NEA", "NET", "PAA", "AFT"), name = "")+
  scale_y_continuous(name = "SESmntd (residuals)", breaks = c(-3, -2, -1,
0, 1, 2, 3, 4))+
  theme(axis.text.y = element_text(size=17))+
  theme(axis.text.x = element_text(size=17))+
  theme(axis.title.y = element_text(size = 17))

herb.veg.mpd <- ggplot(herb, aes(new.veg.type,
model.3.h.mpd.reml$residuals[, 1]))+
  geom_boxplot()+
  theme_classic()+
  scale_x_discrete(labels = c("Closed", "Open"), name = "")+
  scale_y_continuous(name = "SESmpd (residuals)", breaks = c(-8, -6, -4, -
2, 0, 2, 4))+
  theme(axis.text.y = element_text(size=17))+
  theme(axis.text.x = element_text(size=17))+
  theme(axis.title.y = element_text(size = 17))

herb.veg.mntd <- ggplot(herb, aes(new.veg.type,
model.4.h.mntd.reml$residuals[, 1]))+
  geom_boxplot()+
  theme_classic()+
  scale_x_discrete(labels = c("Closed", "Open"), name = "")+
  scale_y_continuous(name = "SESmntd (residuals)", breaks = c(-3, -2, -1,
0, 1, 2, 3, 4))+
  theme(axis.text.y = element_text(size=17))+
  theme(axis.text.x = element_text(size=17))+
  theme(axis.title.y = element_text(size = 17))

# Prepare for high resolution figure
tiff("herb.realm.veg.lat.plot.tiff", units="in", width=15, height=9,
res=600)

# Plot all figures together
ggarrange(herb.realm.mpd, herb.veg.mpd, herb.realm.mntd, herb.veg.mntd,
          labels = c("a)", "b)", "c)", "d)"),
          ncol = 2, nrow = 2)
# Clean graphic environment
dev.off()

#==============================================================================================================================
=

### Correlation tests between latitude and temperature, latitude and
precipitation, and between temperature and precipitation ###

# Woody communities
with(woody, cor.test(latitude, bio1))

with(woody, cor.test(latitude, bio12))

with(woody, cor.test(bio1, bio12))


# Herbaceous communities
with(herb, cor.test(latitude, bio1))

with(herb, cor.test(latitude, bio12))

with(herb, cor.test(bio1, bio12))



###########################################################################################################
#
##########################
##########################
########################## PHYLOGENETIC DIVERSITY IN WOODY COMMUNITIES
WITHOUT GYMNOSPERM SPECIES ##########################
```

```
#########################
#########################
###################################################################################################################
#

#Exclude communities where the calculation of phylogenetic diversity after
#exclude gymnosperm species returned "NA's". They were 15 communities.
#Their removal prior statistica analysis is necessary for plotting partial
residuals.
woody.wg <- subset(woody, woody$ses.mpd.without.gymno != "NA")

### Grouping the data to be analyzed with lme function from the lmer
package ###
group.woody.mpd.wg <- groupedData(ses.mpd.without.gymno ~
latitude|no.article, data = woody.wg)
group.woody.mntd.wg <- groupedData(ses.mntd.without.gymno ~
latitude|no.article, data = woody.wg)

# SESmpd as response variable in woody communities without gymnosperm
species

model.1.w.mpd.wg <- lme(ses.mpd.without.gymno ~ scale(poly(abs.lat, 2)) +
scale(plot.size.ha) + realm,
                        data = group.woody.mpd.wg, random = ~1|no.article,
method = "ML",
                        correlation = corExp(form = ~longitude +
latitude|no.article, nugget = T),
                        na.action = na.exclude)

model.2.w.mpd.wg <- lme(ses.mpd.without.gymno ~ scale(poly(abs.lat, 2)) +
scale(plot.size.ha) + new.veg.type,
                        data = group.woody.mpd.wg, random = ~1|no.article,
method = "ML",
                        correlation = corExp(form = ~longitude +
latitude|no.article, nugget = T),
                        na.action = na.exclude)

model.3.w.mpd.wg <- lme(ses.mpd.without.gymno ~ scale(poly(abs.lat, 2)) +
scale(plot.size.ha) + realm + new.veg.type,
                        data = group.woody.mpd.wg, random = ~1|no.article,
method = "ML",
                        correlation = corExp(form = ~longitude +
latitude|no.article, nugget = T),
                        na.action = na.exclude)

model.4.w.mpd.wg <- lme(ses.mpd.without.gymno ~ scale(poly(abs.lat, 2)) +
scale(plot.size.ha),
                        data = group.woody.mpd.wg, random = ~1|no.article,
method = "ML",
                        correlation = corExp(form = ~longitude +
latitude|no.article, nugget = T),
                        na.action = na.exclude)

# Model selection based on AIC values using MuMIn package
model.sel(model.1.w.mpd.wg, model.2.w.mpd.wg, model.3.w.mpd.wg,
model.4.w.mpd.wg)



# Get marginal and condicional R square using MuMIn package
r.squaredGLMM(model.1.w.mpd.wg)
r.squaredGLMM(model.3.w.mpd.wg)
r.squaredGLMM(model.4.w.mpd.wg)
r.squaredGLMM(model.2.w.mpd.wg)


# Refit the top-ranked model with REML
model.1.w.mpd.reml.wg <- update(model.1.w.mpd.wg, method = "REML")

# Get parameter estimates from the top-ranked model fitted with REML
summary(model.1.w.mpd.reml.wg)

# Get predicted values from the top-ranked model to plot the line
woody$pred.mpd.w.wg <- predict(model.1.w.mpd.reml.wg)

# Plot SESmpd along latitudinal gradient
mpd.without.gymno <- ggplot(woody, aes(abs.lat, ses.mpd.without.gymno)) +
  scale_y_continuous(name="SESmpd",
breaks=pretty(woody$ses.mpd.without.gymno, n = 6))+
  geom_point(alpha = 0.5) +
  theme_classic()+
  scale_x_continuous(name="Latitude (N or S)")+
  geom_lm(mapping = NULL, formula = y ~ x, size = 1, colour = "blue")+
  ggtitle(label = "")+
  theme(plot.title = element_text(hjust = 0.5))+
  theme(axis.text.y = element_text(size=15))+
  theme(axis.text.x = element_text(size=15))




#=====================================================================================================================
=

# SESmntd as response variable in woody communities without gymnosperm
species

model.1.w.mntd.wg <- lme(ses.mntd.without.gymno ~ scale(poly(abs.lat, 2)) +
scale(plot.size.ha) + realm,
```

```
                         data = group.woody.mntd.wg, random =
~1|no.article, method = "ML",
                         correlation = corExp(form = ~longitude +
latitude|no.article, nugget = T),
                         na.action = na.exclude)

model.2.w.mntd.wg <- lme(ses.mntd.without.gymno ~ scale(poly(abs.lat, 2)) +
scale(plot.size.ha) + new.veg.type,
                         data = group.woody.mntd.wg, random =
~1|no.article, method = "ML",
                         correlation = corExp(form = ~longitude +
latitude|no.article, nugget = T),
                         na.action = na.exclude)

model.3.w.mntd.wg <- lme(ses.mntd.without.gymno ~ scale(poly(abs.lat, 2)) +
scale(plot.size.ha) + realm + new.veg.type,
                         data = group.woody.mntd.wg, random =
~1|no.article, method = "ML",
                         correlation = corExp(form = ~longitude +
latitude|no.article, nugget = T),
                         na.action = na.exclude)

model.4.w.mntd.wg <- lme(ses.mntd.without.gymno ~ scale(poly(abs.lat, 2)) +
scale(plot.size.ha),
                         data = group.woody.mntd.wg, random =
~1|no.article, method = "ML",
                         correlation = corExp(form = ~longitude +
latitude|no.article, nugget = T),
                         na.action = na.exclude)




# Model selection based on AIC values using MuMIn package
model.sel(model.1.w.mntd.wg, model.2.w.mntd.wg, model.3.w.mntd.wg,
model.4.w.mntd.wg)

# Get marginal and condicional R square using MuMIn package
r.squaredGLMM(model.4.w.mntd.wg)
r.squaredGLMM(model.2.w.mntd.wg)
r.squaredGLMM(model.1.w.mntd.wg)
r.squaredGLMM(model.3.w.mntd.wg)

# Refit top-ranked model with REML
model.4.w.mntd.wg.reml <- update(model.4.w.mntd.wg, method = "REML")

# Get parameter estimates from the top-ranked model fitted with REML
summary(model.4.w.mntd.wg.reml)

# Get predicted values from the top-ranked model to plot the line
woody$pred.mntd.w.wg <- predict(model.4.w.mntd.wg.reml)

# Plot SESmntd along latitudinal gradient
mntd.without.gymno <- ggplot(woody, aes(abs(latitude),
ses.mntd.without.gymno)) +
  scale_y_continuous(name="SESmntd",
breaks=pretty(woody$ses.mntd.without.gymno, n = 6))+
  geom_point(alpha = 0.5) +
  theme_classic()+
  scale_x_continuous(name="Latitude (N or S)")+
  geom_lm(mapping = NULL, formula = y ~ x, size = 1, colour = "blue")+
  ggtitle(label = "")+
  theme(plot.title = element_text(hjust = 0.5))+
  theme(axis.text.y = element_text(size=15))+
  theme(axis.text.x = element_text(size=15))

# Prepare for high resolution figure
tiff("Phylodiversity.without.gymno.tiff", units="in", width=10, height=5,
res=600)

# Plot all figures together
ggarrange(mpd.without.gymno, mntd.without.gymno,
          labels = c("a)", "b)"),
          ncol = 2, nrow = 1)

# Clean graphic environment
dev.off()



#===========================================================================================================
=

#boxplots realms
tiff("boxplots.realms.without.gymno.tiff", units="in", width=14, height=6,
res=600)

realm.boxplot.mpd.wg <- ggplot(woody.wg, aes(realm,
model.1.w.mpd.reml.wg$residuals[, 1]))+
  geom_boxplot()+
  theme_classic()+
  scale_x_discrete(labels = c("NEA", "NET", "PAA", "AFT", "IN.M", "AUS"),
name = "")+
  scale_y_continuous(name = "SESmpd (residuals)", breaks = c(-4, -2, 0, 2,
4, 6))+
  theme(axis.text.y = element_text(size= 17))+
  theme(axis.text.x = element_text(size= 17))+
  theme(axis.title.y = element_text(size = 17))
```

```
realm.boxplot.mntd.wg <- ggplot(woody.wg, aes(realm,
model.4.w.mntd.wg.reml$residuals[, 1]))+
  geom_boxplot()+
  theme_classic()+
  scale_x_discrete(labels = c("NEA", "NET", "PAA", "AFT", "IN.M", "AUS"),
name = "")+
  scale_y_continuous(name = "SESmntd (residuals)", breaks = c(-4, -2, 0, 2,
4, 6))+
  theme(axis.text.y = element_text(size=17))+
  theme(axis.text.x = element_text(size=17))+
  theme(axis.title.y = element_text(size = 17))

# Plot all figures together
ggarrange(realm.boxplot.mpd.wg, realm.boxplot.mntd.wg,
          labels = c("a)", "b)"),
          ncol = 2, nrow = 1)

# Clean graphic environment
dev.off()

### END ##
```
